# New Glycosylated Secondary Metabolites from Marine-Derived Bacteria

**DOI:** 10.3390/md20070464

**Published:** 2022-07-20

**Authors:** Cao Van Anh, Jong Soon Kang, Hwa-Sun Lee, Phan Thi Hoai Trinh, Chang-Su Heo, Hee Jae Shin

**Affiliations:** 1 Marine Natural Products Chemistry Laboratory, Korea Institute of Ocean Science and Technology, 385 Haeyang-ro, Yeongdo-gu, Busan 49111, Korea; caovananh@kiost.ac.kr (C.V.A.); hwasunlee@kiost.ac.kr (H.-S.L.); science30@kiost.ac.kr (C.-S.H.); 2 Department of Marine Biotechnology, University of Science and Technology (UST), 217 Gajungro, Yuseong-gu, Daejeon 34113, Korea; 3 Laboratory Animal Resource Center, Korea Research Institute of Bioscience and Biotechnology, 30 Yeongudanjiro, Cheongju 28116, Korea; kanjon@kribb.re.kr; 4 Department of Marine Biotechnology, Nhatrang Institute of Technology Research and Application, Vietnam Academy of Science and Technology, 02 Hung Vuong, Nha Trang 650000, Vietnam; phanhoaitrinh84@gmail.com

**Keywords:** marine-derived bacteria, glycosides, pityriacitrin, trehalose lipid, cytotoxicity

## Abstract

Three new glycosylated secondary metabolites, including a new indole alkaloid, pityriacitrin D (**1**), and two new trehalose lipids (**2** and **3**), together with three known compounds (**4**–**6**) were isolated from two marine-derived bacterial strains, *Bacillus siamensis* 168CLC-66.1 and *Tsukamurella pseudospumae* IV19-045. The structures of **1**–**3** were determined by extensive analysis and comparison of their spectroscopic data with literature values. The absolute configurations of sugar moieties were determined by chemical derivatization followed by LC-MS analysis. Cytotoxicity of **1**–**3** against six cancer cell lines was evaluated by SRB assay, and **1** showed moderate activity against all the tested cell lines with GI_50_ values ranging from 8.0 to 10.9 µM.

## 1. Introduction

Bacteria have been recognized as a prolific source of leading structures for new drug development [1,2]. As of early of 2013, nearly 16,000 bacterial natural products (NPs) had been reported, and among them, more than 20% are sugar-based or glycosylated secondary metabolites, containing 344 distinct types of carbohydrates [1]. Drugs based on sugar moieties or glycosides of microbial NPs currently used in clinical application are not uncommon, for example, aminoglycosides [3] or glycosides of macrolides [4] are presently used as anti-bacterial or anti-fungal antibiotics. In addition, anthracycline glycosides are renowned as a class of drugs for the treatment of various cancers [1,5]. Therefore, the discovery of new glycosylated microbial NPs could be an important aspect of pharmaceutical researches.

Among Gram-positive bacteria, the genus *Bacillus* is well-known as a producer of glycosyltrasferases, which can transfer glucose molecules to different types of secondary metabolites, such as macrolactins [6], bacillaenes [7], and ieodoglucomides [8], and these compounds showed various bioactivities, such as anti-microbial, anti-fungal, and cytotoxicity [9]. Additionally, the genus *Tsukamurella* is also known as a bacterial genus producing numerous trehalose lipids, which show biosurfactant activity [10,11,12].

**Figure 1:**
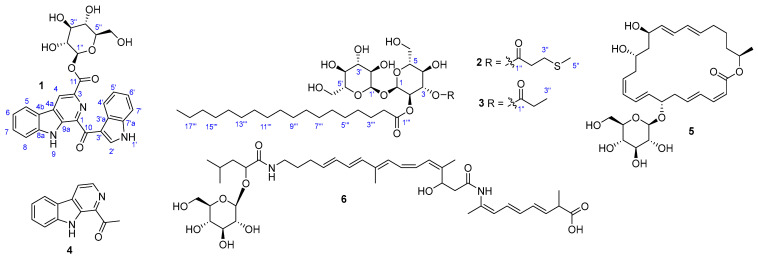
Structures of **1**–**6** isolated from *B. siamensis* 168CLC-66.1 and *T. pseudospumae* IV19-045.

During the course of our research to discover biologically active compounds from marine-derived bacteria, we isolated three new glycosylated NPs (**1**–**3**) from the culture broth of two bacterial strains, *Bacillus siamensis* 168CLC-66.1 and *Tsukamurella pseudospumae* IV19-045, along with three known compounds, 1-acetyl-*β*-carboline (**4**), macrolactin B (**5**), and 12′,13′-*trans*-14′,15′- dihydrobacillaene B (**6**) (Figure 1). Herein, we report the isolation, structure determination, and cytotoxicity of these compounds.

## 2. Results and Discussion

Compound **1** was purified as a yellow amorphous powder. The molecular formula of **1** was determined as C_27_H_23_N_3_O_8_ according to its HRESIMS data (*m*/*z* 540.1381, [M + Na]^+^, cacld. for C_27_H_23_N_3_O_8_Na, 540.1383). The IR spectrum revealed absorption bands at 3339 and 1721 cm^−1^ for hydroxy and ester carbonyl functional groups, respectively. The UV spectrum displayed three maximum absorption bands at 213, 288, and 387 nm, indicating that **1** is an indole alkaloid derivative [13]. The ^1^H NMR spectrum displayed two sets of adjacent aromatic protons at *δ*_H_ 8.21 (d, *J* = 7.7 Hz, H-5), 7.34 (t, *J* = 7.4 Hz, H-6), 7.61 (t, *J* = 7.5 Hz, H-7), and 7.76 (d, *J* = 8.1 Hz, H-8); and 8.57 (d, *J* = 8.5 Hz, H-4′), 7.25 (m, H-5′), 7.25 (m, H-6′), and 7.46 (d, *J* = 8.1 Hz, H-7′), indicating the presence of two ortho-disubstituted benzene rings. Additionally, the ^1^H NMR spectrum also showed two aromatic singlets at *δ*_H_ 9.62 (H-2′) and 8.96 (H-4), and a set of a hexose unit at *δ*_H_ 5.93 (d, *J* = 8.1 Hz, H-1″), 3.76 (t, *J* = 8.5 Hz, H-2″), 3.64 (t, *J* = 8.7 Hz, H-3″), 3.58 (m, H-4″ and H-5″), 3.82 (dd, *J* = 12.3 and 4.6 Hz, H-6″a), and 3.96 (d, *J* = 12.4 Hz, H-6″b). Combination analysis of ^13^C and HSQC NMR data revealed the presence of 21 signals attributed to an aglycone part including a ketocarbonyl at *δ*_C_ 188.4 (C-10), a carbonyl at *δ*_C_ 166.0 (C-11), 10 proton-bearing aromatic carbons, and 9 non-protonated aromatic carbons (Table 1); and 6 signals of a hexose unit at *δ*_C_ 96.7 (C-1″), 74.4 (C-2″), 78.2 (C-3″), 71.2 (C-4″), 79.0 (C-5″), and 62.4 (C-6″). These data indicated that **1** is a glycosylated bisindole alkaloid.

The ^1^H-^1^H COSY spectrum of **1** showed the presence of three spin systems from H-5 to H-8, H-4′ to H-7′, and H-1″ to H-6″. The HMBC correlations from H-5 to C-4a and C-8a; from H-8 to C-4b; and from H-4 to C-5, C-9a, and C-11 identified a substructure of *β*-carboline-3-carboxylic acid. Further, the structure of another indole unit was determined based on the HMBC signals from H-2′ to C-3′, C-3′a, and C-7′a; H-4′/C-7′a; and H-7′/C-3′a. Additionally, H-2′ appeared as a broad singlet at *δ*_H_ 9.62, demonstrating the presence of a 3-acyl-1*H*-indole substructure [13,14]. Based on the aforementioned data and compared with literature data, the aglycone part was determined as pityriacitrin B. A glycosidic linkage between C-1″ and C-11 was confirmed by the HMBC correlation from H-1″ to C-11. The *β*-configuration of the anomeric proton (*δ*_H_ 5.93) was deduced by its coupling constant (*J* = 8.1 Hz). The sugar moiety was determined as d-glucose after acid hydrolysis followed by standard derivatization and HPLC analysis (Figure S25). Therefore, the structure of **1** was determined as a *β*-d-glucopyranosyl derivative of pityriacitrin B and named pityriacitrin D.

Tsukalipid A (**2**) was isolated as a white powder, and its molecular form was determined as C_34_H_62_O_13_S by HRESIMS data (*m*/*z* 733.3804, [M + Na]^+^, calcd. for C_34_H_62_O_13_SNa, 733.3809). The ^1^H NMR spectrum of **2** revealed signals of two methyl groups at *δ*_H_ 0.90 (t, *J* = 7.0 Hz, H_3_-18‴) and 2.10 (s, H_3_-5″); two anomeric protons at *δ*_H_ 5.10 (d, *J* = 3.7 Hz, H-1′) and 5.30 (d, *J* = 3.6 Hz, H-1); two oxymethine ester signals at *δ*_H_ 5.51 (m, H-3) and 4.88 (dd, *J* = 10.3, and 3.7 Hz, H-2); signals of sugar region at *δ*_H_ 3.35–4.01; and methylene signals at *δ*_H_ 1.30–2.73 ppm (Table 2). The ^13^C NMR spectrum, in combination with HSQC data, exhibited signals of two carbonyls at *δ*_C_ 174.6 and 173.1, 12 carbons of two hexanoses at *δ*_C_ 61.9–95.9, 2 methyl groups at *δ*_C_ 15.4 and 14.4, and 18 methylenes at *δ*_C_ 23.7–35.4.

**Figure 2:**
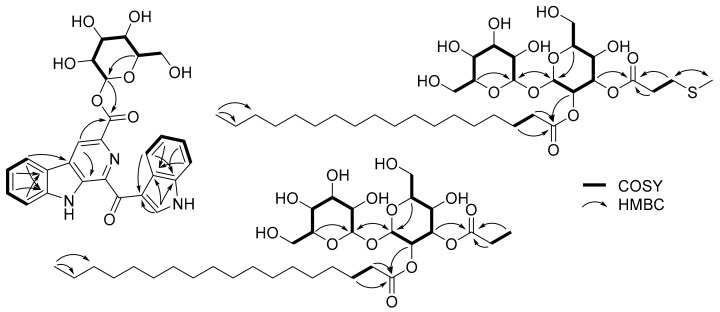
Key 2D NMR correlations of **1**–**3**.

The chemical shifts of H-2 and H-3 were determined by the strong sequential ^1^H-^1^H COSY correlations of H-1/H-2/H-3 (*δ*_H_ 5.30/4.88/5.51). A partial structure of 3-(methylthio)propanoic acid was determined by the HMBC correlations of H_2_-2″/C-1″, H_2_-2″/C-3″, H_2_-3″/C-1″, H_2_-3″/C-2″, H_2_-3″/C-5″, and H_3_-5″/C-3″, and the connection of this acid to sugar moiety at C-3 was corroborated by the strong HMBC correlation from H-3 to C-1″. Moreover, the HMBC correlations from H-1 to C-1′ (5.30 to 95.9) and H-1′ to C-1 (5.10 to 93.0) indicated two sugars were connected via a (1→1)-glycosidic linkage. Further, 18 remaining carbons were assigned as stearic acid by the continuous ^1^H-^1^H COSY correlations from H_2_-2‴ to H_3_-18‴, and the HMBC correlations from H_2_-2‴ and H_2_-3‴ to C-1‴, and the connection of stearic acid with the sugar moiety at C-2 via an ester bond was confirmed by the HMBC correlation from H-2 to C-1‴ (Figure 2). Two anomeric protons observed with small coupling constants (*J* = 3.6 and 3.7 Hz), indicated they have an *α*-configuration, and both of them were determined as d-glucose by a similar procedure for **1**. Therefore, the structure of **2** was determined as depicted in Figure 1. A literature search revealed that many trehalose lipids were isolated from the genus *Tsukamurella* [10,11,12], however, **2** was the first example of sulfur-containing trehalose lipids isolated from this genus.

Tsukalipid B (**3**) was isolated as a white powder, and the molecular formula of **3** was determined as C_33_H_60_O_13_ based on its HRESIMS peak at *m*/*z* 687.3931, [M + Na]^+^ (calcd. for C_33_H_60_O_13_Na, 687.3932). The 1D and 2D NMR data of **3** quite resembled those of **2**, except for the terminal methyl of the short-chain carboxylic acid observed at *δ*_H_ 1.12 (t, *J* = 7.6 Hz, H_3_-3″). Further, this acid was determined as propanoic acid by the HMBC correlations from H_3_-3″ and H_2_-2″ to C-1″. The sugar moiety and the long-chain fatty acid were determined to be the same as that of **2** (trehalose and stearic acid) by a similar procedure for **2**. Thus, the structure of **3** was determined as shown in Figure 1.

The structures of the known compounds were identified as 1-acetyl-*β*-carboline (**4**), macrolactin B (**5**), and 12′,13′-*trans*-14′,15′-dihydrobacillaene B (**6**) by comparison of their spectroscopic data with those reported in literature [7,15,16].

Since many bacterial glycosylated secondary metabolites showed in-vitro cytotoxic activity, the new compounds (**1**–**3**) were preliminarily screened for their cytotoxicity against six cancer cell lines, which are among the most common cancers in Korea (Table 3). Compound **1** showed moderate activity with GI_50_ values ranging from 8.0 to 10.9 μM. The result indicated that glycosylated natural products isolated from *Bacillus* spp. could be an attractive source for the discovery of new anti-cancer leads. Compounds **2** and **3** did not show significant cytotoxicity at a concentration of 30 µg/mL. Even though, many trehalose lipid isolated from the genus *Tsukamurella* showed biosurfactant activity, due to the limited amount of **2** and **3**, it was unable to check their effect on surface and interfacial tension. Therefore, further studies are needed to find bioactivities of **2** and **3**.

## 3. Materials and Methods

### 3.1. General Experimental Procedures

HRESIMS data were recorded using a Waters Synapt G2 Q-TOF mass spectrometer (Waters Corporation, Milford, MA, USA). Optical rotations were obtained on a Rudolph Research Analytical Autopol III polarimeter (Rudolph Research Analytical, Hackettstown, NJ, USA). 1D and 2D NMR spectra were measured using a Bruker 600 MHz spectrometer (Bruker BioSpin GmbH, Rheinstetten, Germany). IR spectra were recorded using a JASCO FT/IR-4100 spectrophotometer (JASCO Corporation, Tokyo, Japan). UV-visible spectra were obtained by a Shimadzu UV-1650PC spectrophotometer. HPLC was performed with a PrimeLine Binary pump (Analytical Scientific Instruments, Inc., El Sobrante, CA, USA) and a RI-101 detector (Shoko Scientific Co. Ltd., Yokohama, Japan). Semi-preparative HPLC was carried out using an ODS column (YMC-Pack-ODS-A, 250 × 10 mm i.d., 5 μM). Analytical HPLC was conducted with an ODS column (YMC-Pack-ODS-A, 250 × 4.6 mm i.d., 5 μM). All reagents were obtained from Sigma-Aldrich (Merck KGaA, Darmstadt, Germany).

### 3.2. Bacterial Strain, Fermentation, and Isolation of ***1*** and ***4***–***6*** from Bacillus siamensis 168CLC-66.1

The strain 168CLC-66.1 was isolated from a seaweed sample (*Caulerpa* sp.) collected near Cu Lao Cham peninsula, Vietnam in August 2016. The strain was identified as *Bacillus siamensis* on the basis of 16S rRNA gene sequence analysis (GenBank accession number ON631768). The seed and mass cultures of the strain were carried out in Bennett’s medium (BN broth, 1% glucose, 0.1% yeast extract, 0.2% tryptone, 0.1% beef extract, 0.5% glycerol, and 3.2% sea salt). The strain was cultured in 50 mL of the BN medium in a 100 mL Erlenmeyer flask. After 4 days of cultivation on a rotary shaker at 120 rpm and 28 °C, 20 mL of the culture medium was inoculated into 1.0 L of the BN medium in a 2.0 L Erlenmeyer flask under the same conditions for 4 days, and then the culture broth was incubated into a 100 L fermenter filled with 70 L of BN medium and cultured for 7 days and then harvested. The culture was separated into the cells and broth by centrifugation, and the broth was extracted with EtOAc (70 L, twice). The EtOAc layer was evaporated to yield a crude extract (5.0 g). The extract was separated into 15 fractions (F1 to F15) by vacuum liquid chromatography on an ODS column using a stepwise elution with 3 × 300 mL each of 20%, 40%, 60%, 80% MeOH in H_2_O, and 100% MeOH. The F10 fraction was purified by a semipreparative HPLC (YMC-PackODS-A, 250 × 10 mm i.d., 5 μm, flow rate 2.0 mL/min) with an isocratic elution of 65% MeOH in H_2_O to yield compounds **4** (3.0 mg, *t_R_* = 14.2 min) and **5** (2.0 mg, *t_R_* = 16.5 min). Compounds **1** (3.0 mg, *t_R_* = 16.0 min) and **6** (3.5 mg, *t_R_* = 21.1 min) were isolated from the F11 fraction using a semipreparative HPLC (YMC-PackODS-A, 250 × 10 mm i.d., 5 μm, flow rate 2.0 mL/min) with an isocratic elution of 70% MeOH in H_2_O.

Pityriacitrin D (**1**): yellow powder, ${\left[\alpha \right]}_{D}^{20}$ −10 (*c* 0.1, MeOH); IR ν_max_ 3339, 2925, 2851, 1721, 1596, 1437, 1339 cm^−1^, UV(MeOH) λ_max_ (log ε) 213 (4.0), 288 (3.6), 387 (3.3) nm, HRESIMS *m*/*z* 540.1381 [M + Na]^+^ (calcd. for C_27_H_23_N_3_O_8_Na, 540.1383), ^1^H NMR (CD_3_OD, 600 MHz) and ^13^C NMR (CD_3_OD, 150 MHz) see Table 1.

### 3.3. Bacterial Strain, Fermentation, and Isolation of ***2*** and ***3*** from Tsukamurella pseudospumae IV19-045

The strain IV19-045 was isolated from a sediment sample collected using a multi-corer (MC) mounted on the R/V ISABU from the Indian Ocean in June 2019 (latitude: 11°14′56.4″ S, longtitude: 66°15′14.4″ E, depth: 2020 m). The strain IV19-045 was identified as *Tsukamurella pseudospumae* on the basis of 16S rRNA gene sequence analysis (GenBank accession number ON545811). The seed and mass cultures were conducted by the same procedure described for *B. siamensis* 168CLC.66.1. After 7 days, the culture was separated into the cells and supernatant by centrifugation. The cells were extracted with MeOH (5 L, twice) and the solvent was concentrated under reduced pressure to yield a crude extract (5.0 g), which was fractionated into 10 fractions (M1 to M10) by an ODS column using a stepwise elution of 10–100% MeOH in H_2_O. The M10 fraction was purified by a semipreparative HPLC (YMC-PackODS-A, 250 × 10 mm i.d., 5 μm, flow rate 2.0 mL/min) with an isocratic elution of 90% MeOH in H_2_O to obtain compounds **2** (1.5 mg, *t_R_* = 39.5 min) and **3** (1.5 mg, *t_R_* = 33.0 min).

Tsukalipid A (**2**): white solid, ${\left[\alpha \right]}_{D}^{20}$ +90 (*c* 0.05, MeOH); IR *ν*_max_ 3364, 2922, 2851, 1738, 1458, 1152 cm^−1^, HRESIMS *m*/*z* 733.3804, [M + Na]^+^ (calcd. for C_34_H_62_O_13_SNa, 733.3809), ^1^H NMR (CD_3_OD, 600 MHz) and ^13^C NMR (CD_3_OD, 150 MHz) see Table 2.

Tsukalipid B (**3**): white solid, ${\left[\alpha \right]}_{D}^{20}$ +95 (*c* 0.05, MeOH); IR *ν*_max_ 3360, 2922, 2855, 1738, 1462, 1352, 1152 cm^−1^, HRESIMS *m*/*z* 687.3931, [M + Na]^+^ (calcd. for C_33_H_60_O_13_Na, 687.3932), ^1^H NMR (CD_3_OD, 600 MHz) and ^13^C NMR (CD_3_OD, 150 MHz) see Table 2.

### 3.4. Acid Hydrolysis and Determination of the Absolute Configuration of Glucose

Compound **1** (1.0 mg) was dissolved in 3 N HCl (1.0 mL) and heated to 80 °C for 1 h. The solution was cooled and extracted twice with EtOAc. The aqueous layer was dried to give the sugar residue. The residue was dissolved in pyridine (0.5 mL) containing l-cysteine methyl ester hydrochloride (0.5 mg) and heated to 60 °C for 1 h. *o*-Tolylisothiocyanate (10 μL) was added to the mixture, and heating was continued for an additional 1 h. The reaction mixture was directly analyzed using HPLC (gradient elution of 10% to 100% MeCN in H_2_O over 50 min). The sugar residue of **1** was detected at 18.17 min. The retention times of the authentic glucose samples were 18.17 (d-glucose) and 17.86 (l-glucose) min under the same HPLC conditions. Therefore, the absolute configuration of the glucose unit in **1** was established as d-glucose. The sugar moieties of **2** and **3** were also determined as d-glucose by the same procedure described for **1**.

### 3.5. Cytotoxicity Test by SRB Assay

The SRB cytotoxicity test for **1**–**3** was conducted as previously described [17]. One-way ANOVA followed by Dunnett’s *t*-test was used for statistical analysis and the GI_50_ values were determined by the software of GraphPad Prism 8 (GraphPad Software Inc., San Diego, CA, USA). Cancer cell lines were purchased from Japanese Cancer Research Resources Bank (JCRB) (NUGC-3, JCRB Cell Bank/Cat. #JCRB0822) and American Type Culture Collection (ATCC) (PC-3, ATCC/Cat. #CRL-1435; MDA-MB-231, ATCC/Cat. #HTB-26; ACHN, ATCC/Cat. #CRL-1611; NCI-H23, ATCC/Cat. #CRL-5800; HCT-15, ATCC/Cat. #CCL-225).

## 4. Conclusions

In conclusion, we isolated three new glycosylated secondary metabolites from two marine-derived bacterial strains, *Bacillus siamensis* 168CLC-66.1 and *Tsukamurella pseudospumae* IV19-045, including a new indole alkaloid (**1**) and two new trehalose lipids (**2** and **3**). The structures of the new compounds were determined by spectroscopic methods (HRESIMS, 1D and 2D NMR). The sugar units were determined by acidic hydrolysis followed by chemical derivatization and LC-MS analysis. Compound **2** is the first example of sulfur-containing trehalose lipids isolated from the genus *Tsukamurella*. In-vitro cytotoxicity of **1**–**3** against a panel of cancer cell lines was evaluated, and **1** showed moderate activity with GI_50_ values of 8.0–10.9 µM. These results enriched biochemical diversities of microbial glycosylated natural products, which could be used for further researches to find new anti-cancer leads.

## Figures and Tables

**Table 1:** ^1^H and ^13^C NMR spectroscopic data of **1** (600 MHz for ^1^ H and 150 MHz for ^13^C, CD_3_OD).

	*δ*_H_, Mult (*J* in Hz)	*δ*_C_, Type		*δ*_H_, Mult (*J* in Hz)	*δ*_C_, Type
1		135.7, C	2′	9.62, s	140.1, CH
3		139.0, C	3′		115.9, C
4	8.96, s	121.1, CH	3′a		128.9, C
4a		132.8, C	4′	8.57, d (8.5)	122.7, CH
4b		124.1, C	5′	7.25, ovl	123.4, CH
5	8.21, d (7.7)	122.2, CH	6′	7.25, ovl	123.3, CH
6	7.34, t (7.4)	122.2, CH	7′	7.46, d (8.1)	112.8, CH
7	7.61, t (7.5)	130.4, CH	7′a		137.5, C
8	7.76, d (8.1)	113.9, CH	1″	5.93, d (8.1)	96.7, CH
8a		143.4, C	2″	3.76, t (8.5)	74.4, CH
9a		138.1, C	3″	3.64, t (8.7)	78.2, CH
10		188.4, C	4″	3.58, m	71.2, CH
11		166.0, C	5″	3.58, m	79.0, CH
			6″	3.96, d (12.4) 3.82, dd (12.3, 4.6)	62.4, CH_2_

**Table 2:** ^1^H and ^13^C NMR spectroscopic data of **2** and **3** (600 MHz for ^1^H and 150 MHz for ^13^C, CD_3_OD).

	2		3	
	*δ*_H_, Mult (*J* in Hz)	*δ*_C_, Type	*δ*_H_, Mult (*J* in Hz)	*δ*_C_, Type
1	5.30, d (3.6)	93.0, CH	5.30, d (3.6)	93.0, CH
2	4.88, dd (10.3, 3.7)	71.9, CH	4.87, dd (10.3, 3.7)	72.1, CH
3	5.51, m	74.3, CH	5.49, m	73.9, CH
4	3.63, m	69.6, CH	3.63, m	69.6, CH
5	4.01, ddd (10.0, 4.6, 2.2)	73.7, CH	4.01, ddd (10.0, 4.6, 2.2)	73.7, CH
6	3.82, m 3.81, m	61.9, CH_2_	3.82, m 3.73, m	62.0, CH_2_
1′	5.10, d (3.7)	95.9, CH	5.10, d (3.7)	95.9, CH
2′	3.49, dd (9.8, 3.8)	73.0, CH	3.50, dd (9.8, 3.7)	73.0, CH
3′	3.77, m	74.6, CH	3.78, m	74.6, CH
4′	3.35, m	71.6, CH	3.34, dd (12.2, 6.1)	71.6, CH
5′	3.66, m	74.2, CH	3.66, m	74.3, CH
6′	3.70, m 3.66, m	62.5, CH_2_	3.71, m 3.66, m	62.4, CH_2_
1″		173.1, C		175.5, C
2″	2.65, m	35.4, CH_2_	2.36, m	28.4, CH_2_
3″	2.73, m	29.9, CH_2_	1.12, t (7.6)	9.5, CH_3_
5″	2.10, s	15.4, CH_3_		
1‴		174.6, C		174.4, C
2‴	2.37, m	35.0, CH_2_	2.34, m	34.9, CH_2_
3‴	1.57, m	25.8, CH_2_	1.56, m	25.9, CH_2_
4‴	1.30, ovl	30.3, CH_2_	1.30, ovl	30.2, CH_2_
5‴	1.30, ovl	30.5, CH_2_	1.30, ovl	30.4, CH_2_
6‴	1.30, ovl	30.6, CH_2_	1.30, ovl	30.5, CH_2_
7‴	1.30, ovl	30.8, CH_2_	1.30, ovl	30.6, CH_2_
8‴	1.30, ovl	30.8, CH_2_	1.30, ovl	30.7, CH_2_
9‴	1.30, ovl	30.8, CH_2_	1.30, ovl	30.8, CH_2_
10‴	1.30, ovl	30.8, CH_2_	1.30, ovl	30.8, CH_2_
11‴	1.30, ovl	30.8, CH_2_	1.30, ovl	30.8, CH_2_
12‴	1.30, ovl	30.8, CH_2_	1.30, ovl	30.8, CH_2_
13‴	1.30, ovl	30.8, CH_2_	1.30, ovl	30.8, CH_2_
14‴	1.30, ovl	30.8, CH_2_	1.30, ovl	30.8, CH_2_
15‴	1.30, ovl	30.8, CH_2_	1.30, ovl	30.8, CH_2_
16‴	1.30, ovl	33.1, CH_2_	1.30, ovl	33.1, CH_2_
17‴	1.30, ovl	23.7, CH_2_	1.30, ovl	23.7, CH_2_
18‴	0.90, t (7.0)	14.4, CH_3_	0.90, t (7.0)	14.4, CH_3_

**Table 3:** Growth inhibition (GI_50_, μM) values of **1** against six human cancer cell lines.

Cell Line ^a^	GI_50_, μM	ADR ^b^
HCT-15	10.9	0.13
NUGC-3	9.8	0.16
NCI-H23	10.2	0.14
ACHN	10.1	0.17
PC-3	8.8	0.17
MDA-MB-231	8.0	0.16

^a^ HCT-15: colon cancer, NUGC-3: stomach cancer, NCI-H23: lung cancer, ACHN: renal cancer, PC-3: prostate cancer, MDA-MB-231: breast cancer; GI_50_ values are the concentration corresponding to 50% growth inhibition. ^b^ ADR; adriamycin as standard.

## Data Availability

The data presented in the article are available in the Supplementary Materials.

## Supplementary Materials

The following are available online at https://www.mdpi.com/article/10.3390/md20070464/s1, Figures S1–S24. 1D and 2D NMR experimental spectra and HRESIMS data of compounds **1**–**3**. Figure S25. Analysis of sugar moieties. Figure S26. Results of the cytotoxicity test of **1**. Figures S27–S29, ^1^H NMR spectra of compounds **4**–**6**.
