# Supplementary material for: New Glycosylated Secondary Metabolites from Marine-Derived Bacteria
Source: Mar Drugs. 2022 Jul 20;20(7):464. doi: 10.3390/md20070464 (PMC9321207; doi:10.3390/md20070464)
Supplement: Supplementary file 1 [file marinedrugs-20-00464-s001.zip › marinedrugs-1805509-supplementary.pdf]

Article

New Glycosylated Secondary Metabolites from Marine-Derived Bacteria

Cao Van Anh [1,2], Jong Soon Kang [3], Hwa-Sun Lee [1], Phan Thi Hoai Trinh [4], Chang-Su Heo [1,2] and Hee Jae Shin [1,2,*]

[1] Marine Natural Products Chemistry Laboratory, Korea Institute of Ocean Science and Technology, 385 Haeyang-ro, Yeongdo-gu, Busan 49111, Korea; caovananh@kiost.ac.kr (C.V.A.); hwasunlee@kiost.ac.kr (H.-S.L.); science30@kiost.ac.kr (C.-S.H.)

[2] Department of Marine Biotechnology, University of Science and Technology (UST), 217 Gajungro, Yuseong-gu, Daejeon 34113, Korea

[3] Laboratory Animal Resource Center, Korea Research Institute of Bioscience and Biotechnology, 30 Yeongudanjiro, Cheongju 28116, Korea; kanjon@kribb.re.kr

[4] Department of Marine Biotechnology, Nhatrang Institute of Technology Research and Application, Vietnam Academy of Science and Technology, 02 Hung Vuong, Nha Trang 650000, Vietnam; phanhoaitrinh84@gmail.com

* Correspondence: shinhj@kiost.ac.kr; Tel.: +82-51-664-3341; Fax: +82-51-664-3340

Contents

Figure S1. ^{1}H NMR spectrum of **1** (CD_3OD, 600 MHz). 3
Figure S2. ^{13}C NMR spectrum of **1** (CD_3OD, 150 MHz). 3
Figure S3. HSQC spectrum of **1** (CD_3OD). 4
Figure S4. ^{1}H-^{1}H COSY spectrum of **1** (CD_3OD). 5
Figure S5. HMBC spectrum of **1** (CD_3OD). 6
Figure S5. HMBC spectrum of **1** (CD_3OD) (extended H-4). 7
Figure S6. ^{1}H NMR spectrum of **1** (DMSO-*d6*, 600 MHz). 8
Figure S7. ^{13}C NMR spectrum of **1** (DMSO-*d6*, 150 MHz). 8
Figure S8. HSQC spectrum of **1** (DMSO-*d6*). 9
Figure S9. ^{1}H-^{1}H COSY spectrum of **1** (DMSO-*d6*). 10
Figure S10. HMBC spectrum of **1** (DMSO-*d6*). 11
Figure S11. NOESY spectrum of **1** (DMSO-*d6*). 12
Figure S12. HRESIMS data of **1**. 13
Figure S13. ^{1}H NMR spectrum of **2** (CD_3OD, 600 MHz). 14
Figure S14. ^{13}C NMR spectrum of **2** (CD_3OD, 150 MHz). 15
Figure S15. HSQC spectrum of **2** (CD_3OD). 16
Figure S16. ^{1}H-^{1}H COSY spectrum of **2** (CD_3OD). 17
Figure S17. HMBC spectrum of **2** (CD_3OD). 18
Figure S18. HRESIMS data of **2**. 19
Figure S19. ^{1}H NMR spectrum of **3** (CD_3OD, 600 MHz). 20
Figure S20. ^{13}C NMR spectrum of **3** (CD_3OD, 150 MHz). 20
Figure S21. HSQC spectrum of **3** (CD_3OD). 21
Figure S22. ^{1}H-^{1}H COSY spectrum of **3** (CD_3OD). 22
Figure S23. HMBC spectrum of **3** (CD_3OD). 23
Figure S24. HRESIMS data of **3**. 24
Figure S25. Analysis of sugar moieties. 25
Figure S26. Results of the cytotoxicity test of **1**. 26
Figure S27. ^{1}H NMR spectrum of **4** (DMSO-d_6, 600 MHz). 27
Figure S28. ^{1}H NMR spectrum of **5** (CD_3OD, 600 MHz). 27
Figure S29. ^{1}H NMR spectrum of **6** (CD_3OD, 600 MHz). 28

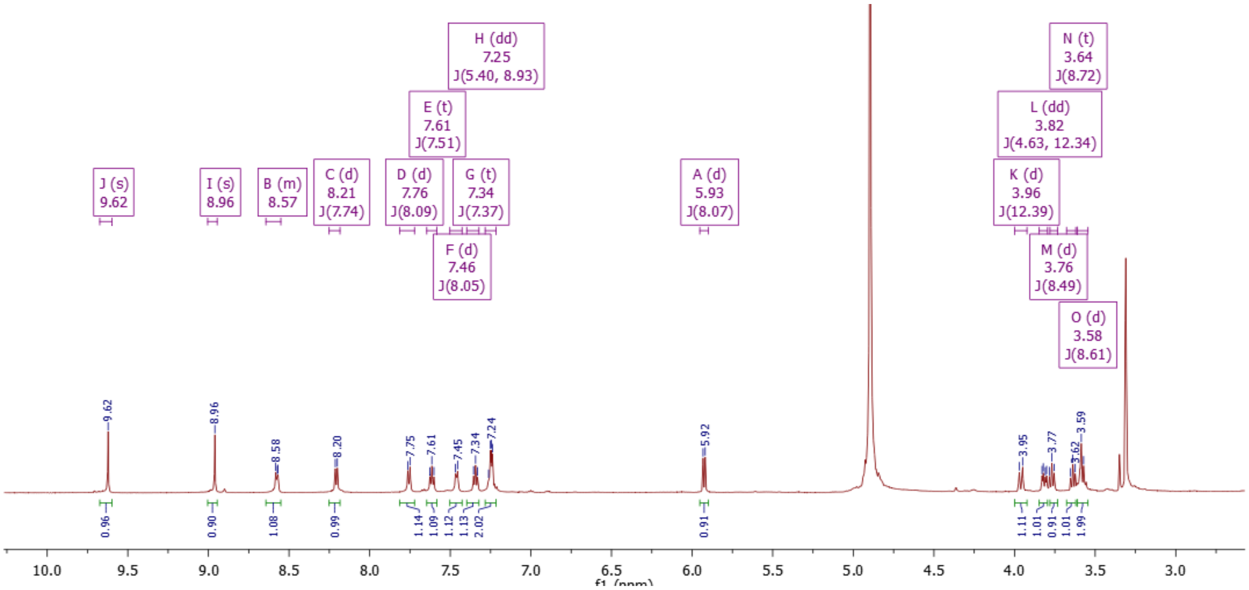

Figure S1. ^{1}H NMR spectrum of **1** (CD3OD, 600 MHz).

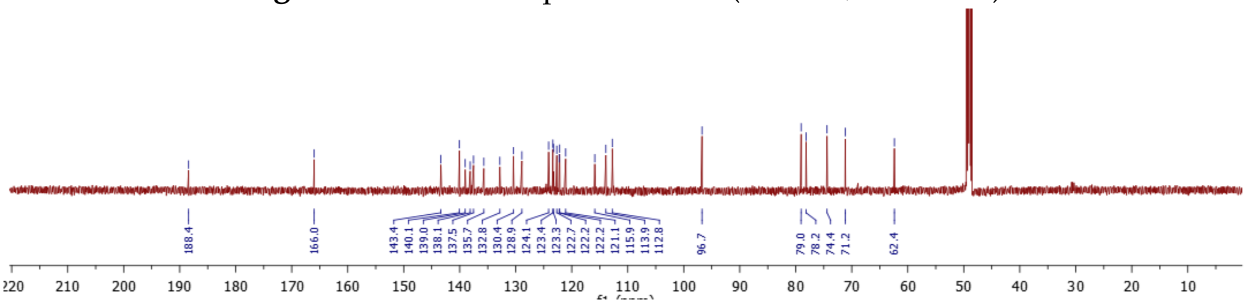

Figure S2. ^{13}C NMR spectrum of **1** (CD3OD, 150 MHz).

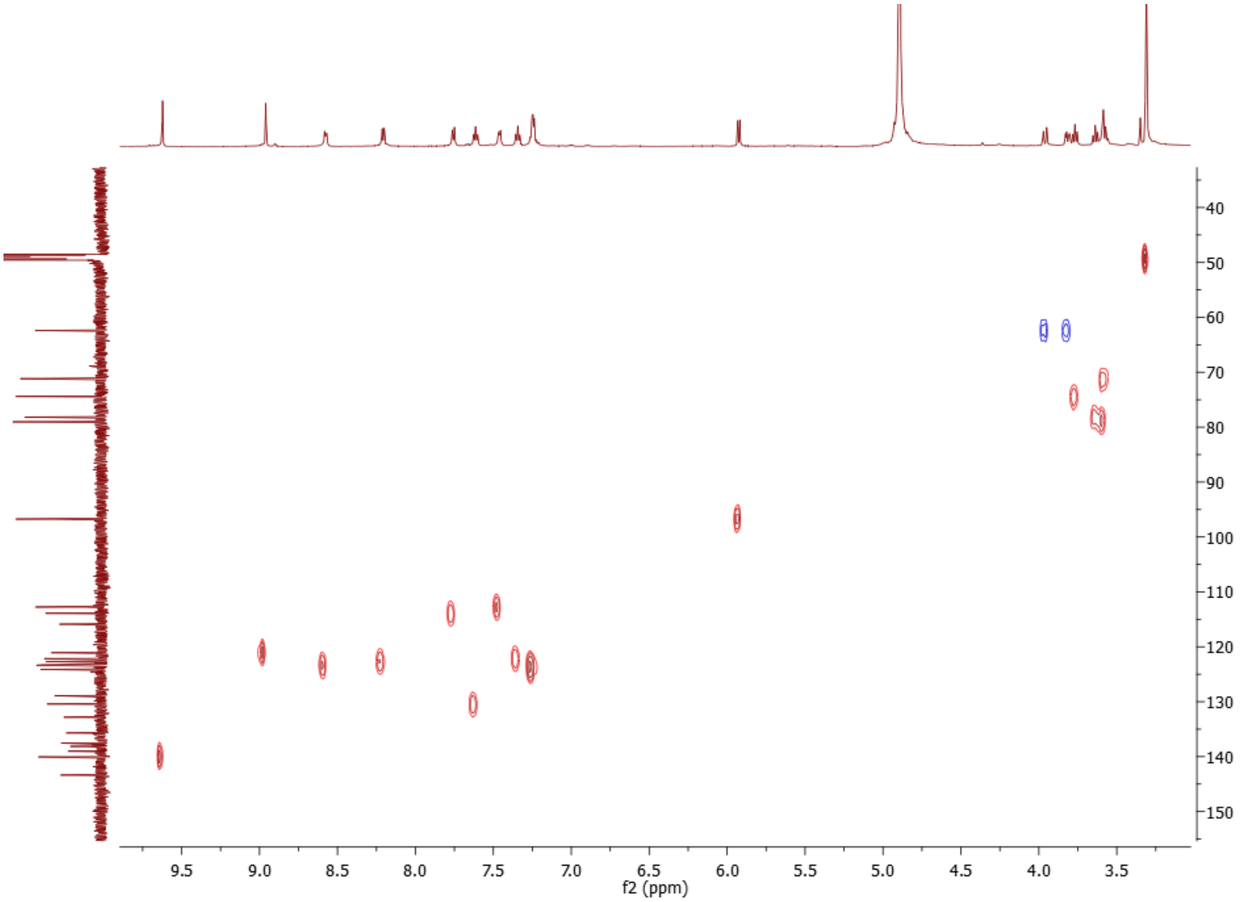

Figure S3. HSQC spectrum of **1** (CD_3OD).

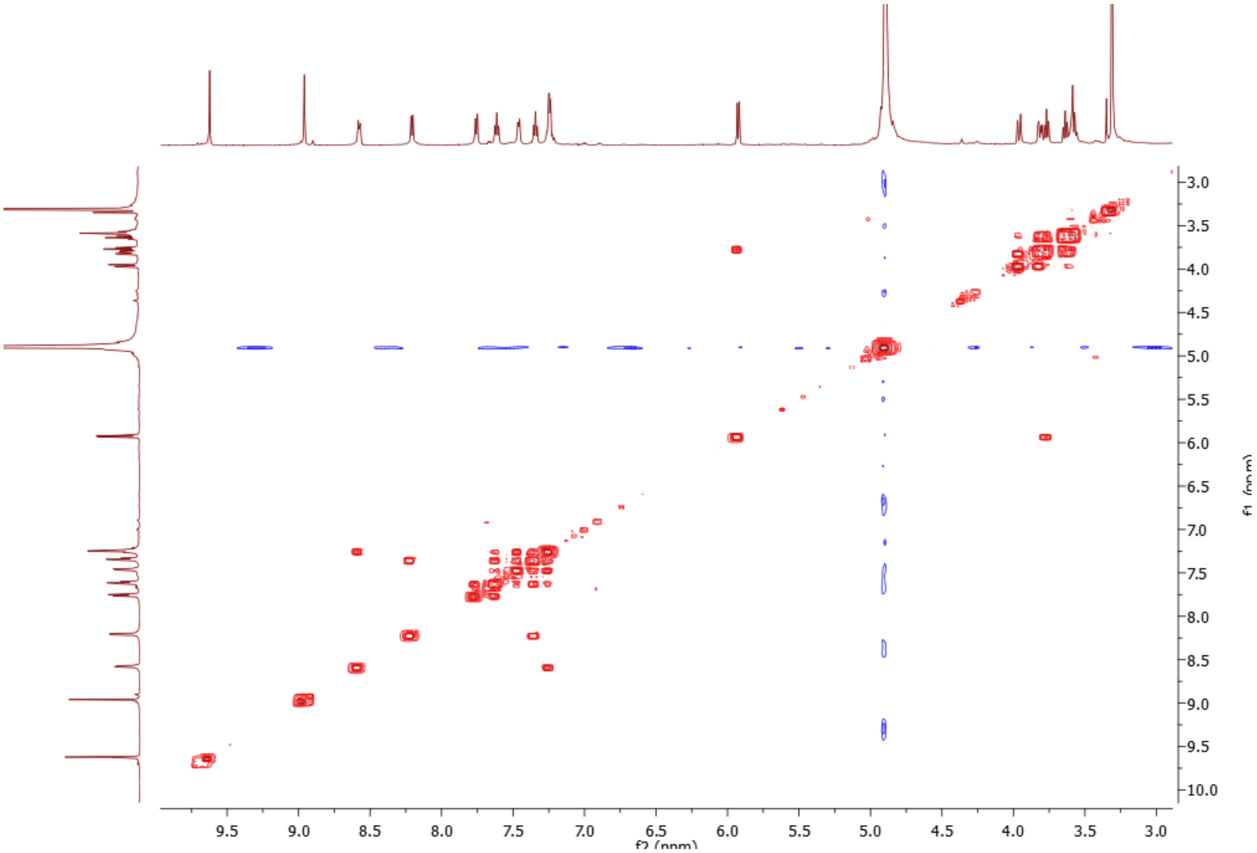

Figure S4. ^{1}H-^{1}H COSY spectrum of **1** (CD_3OD).

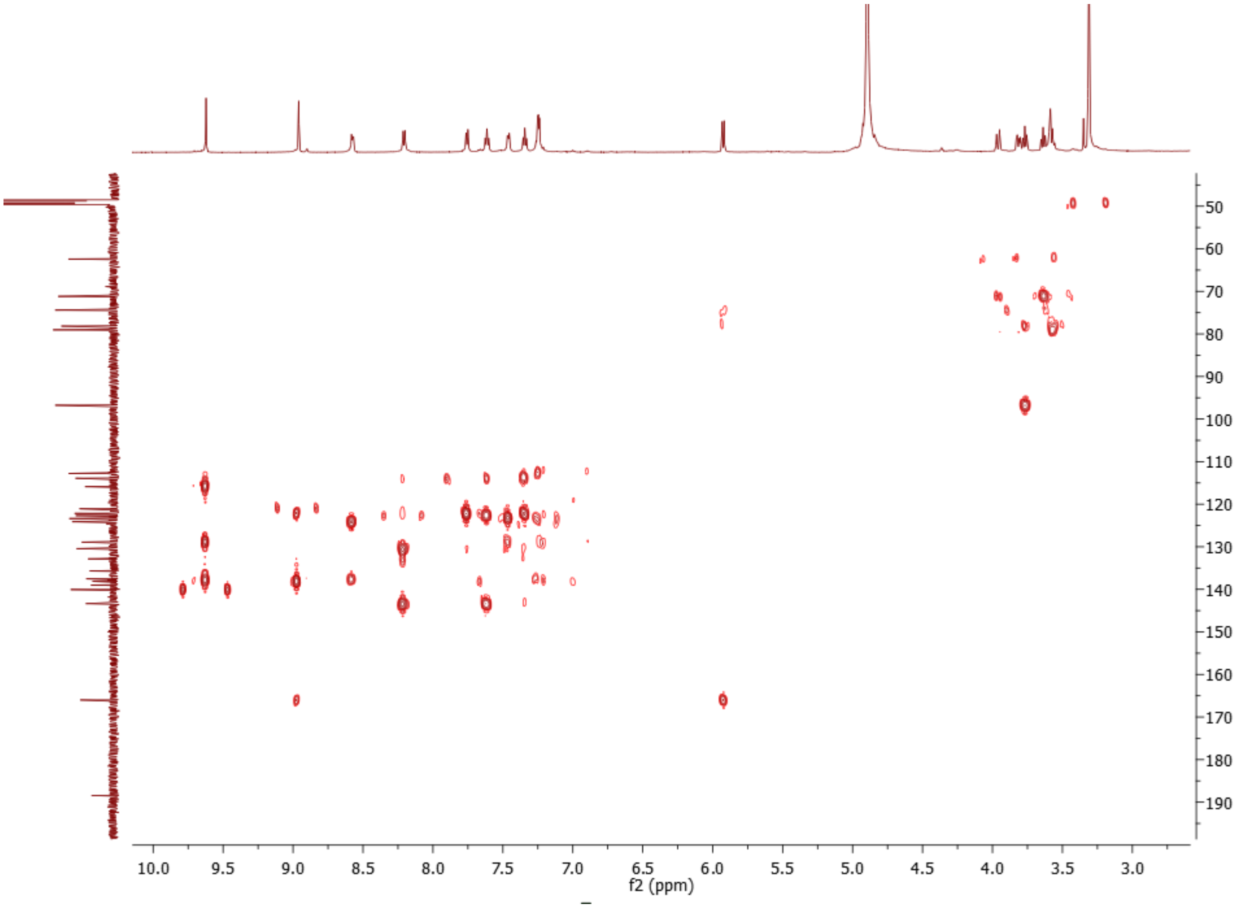

Figure S5. HMBC spectrum of **1** (CD_3OD).

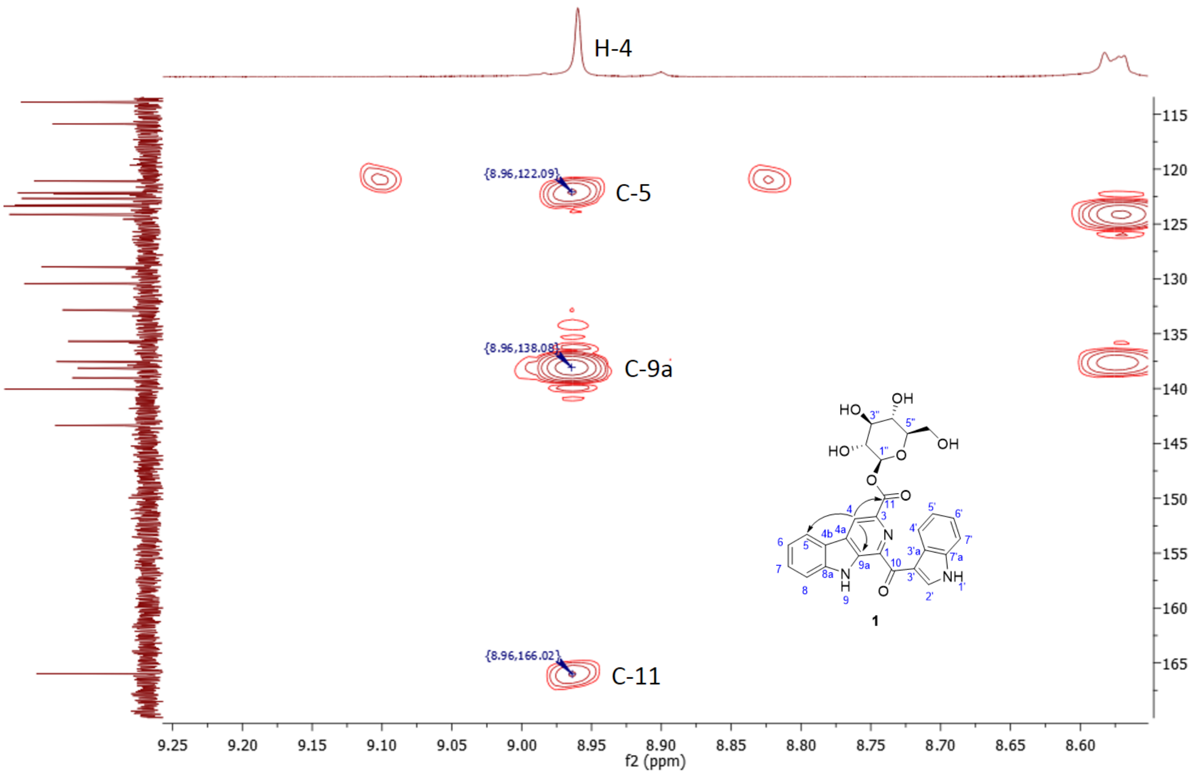

Figure S5. HMBC spectrum of **1** (CD_3OD) (extended H-4).

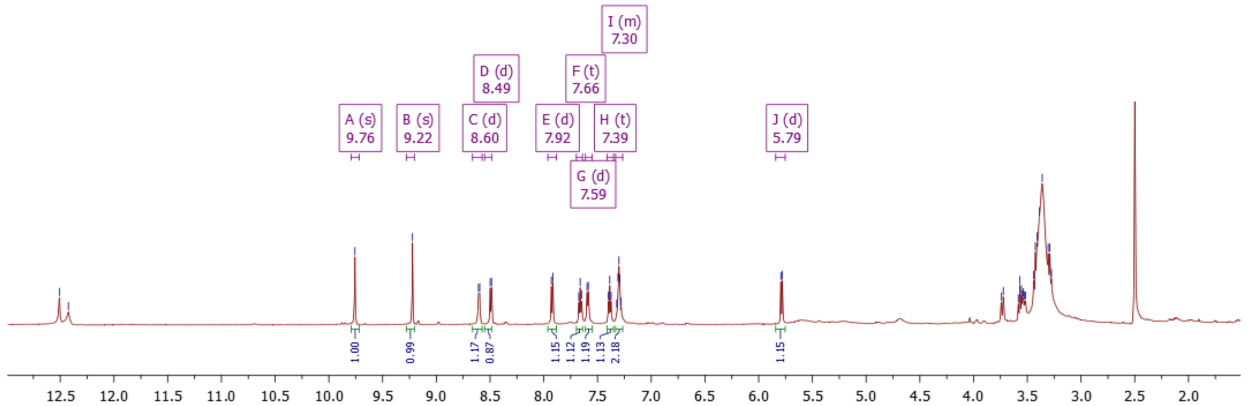

Figure S6. ^{1}H NMR spectrum of **1** (DMSO-*d6*, 600 MHz).

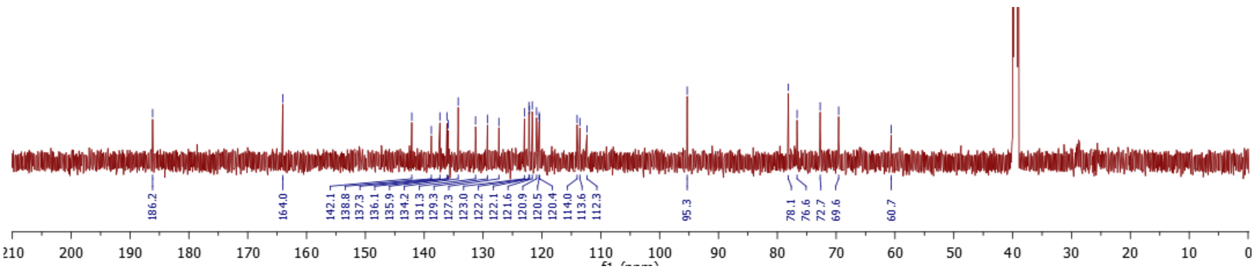

Figure S7. ^{13}C NMR spectrum of **1** (DMSO-*d6*, 150 MHz).

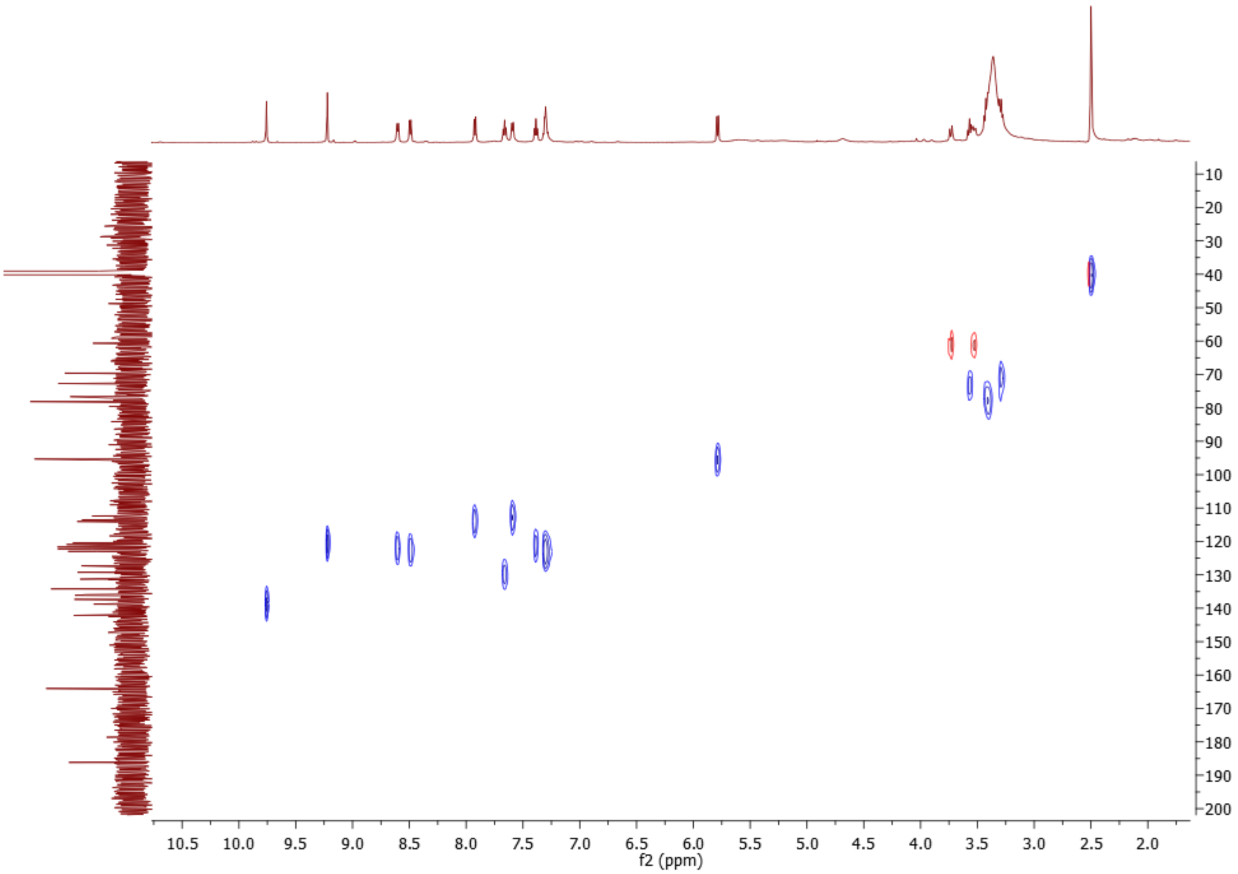

Figure S8. HSQC spectrum of **1** (DMSO-*d6*).

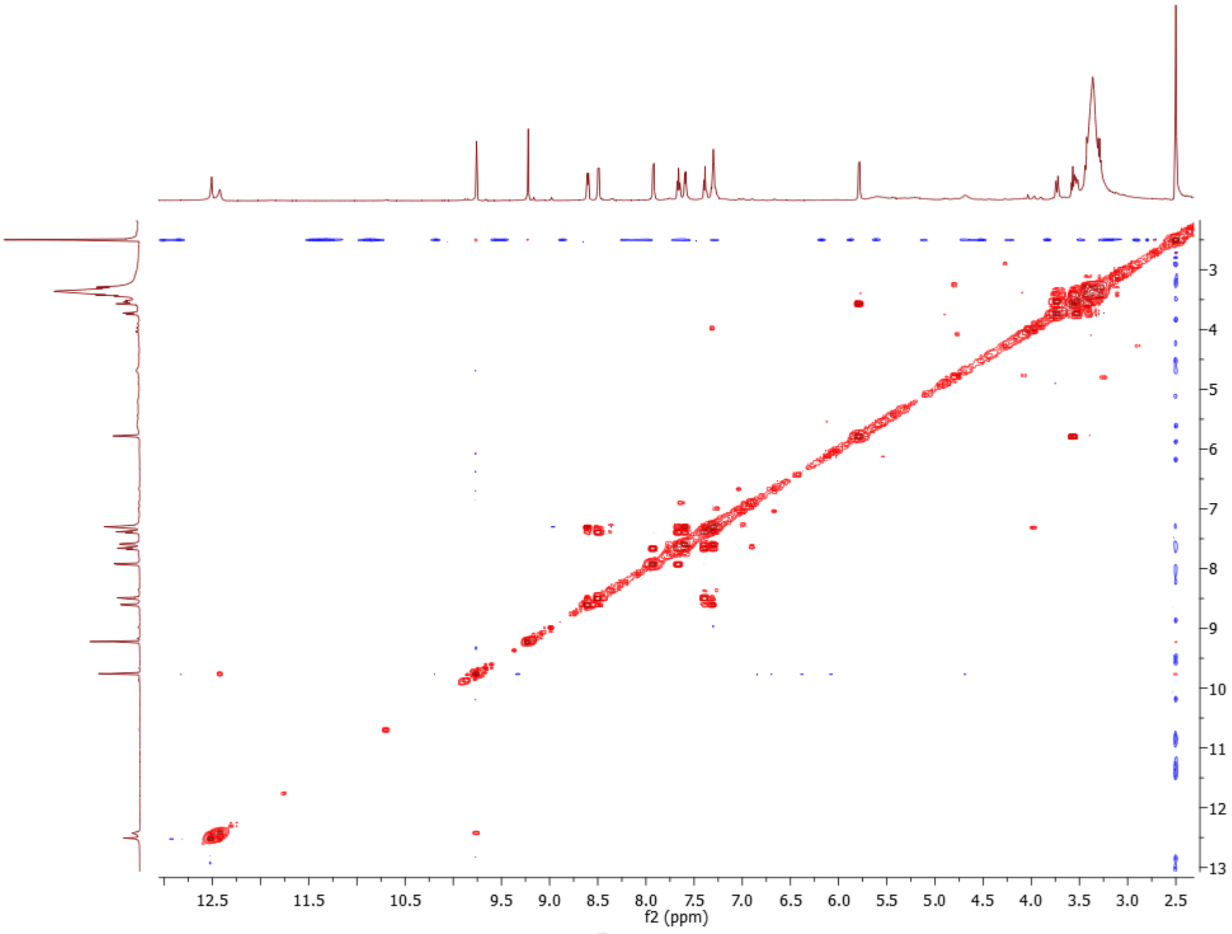

Figure S9. ^{1}H-^{1}H COSY spectrum of **1** (DMSO-*d6*).

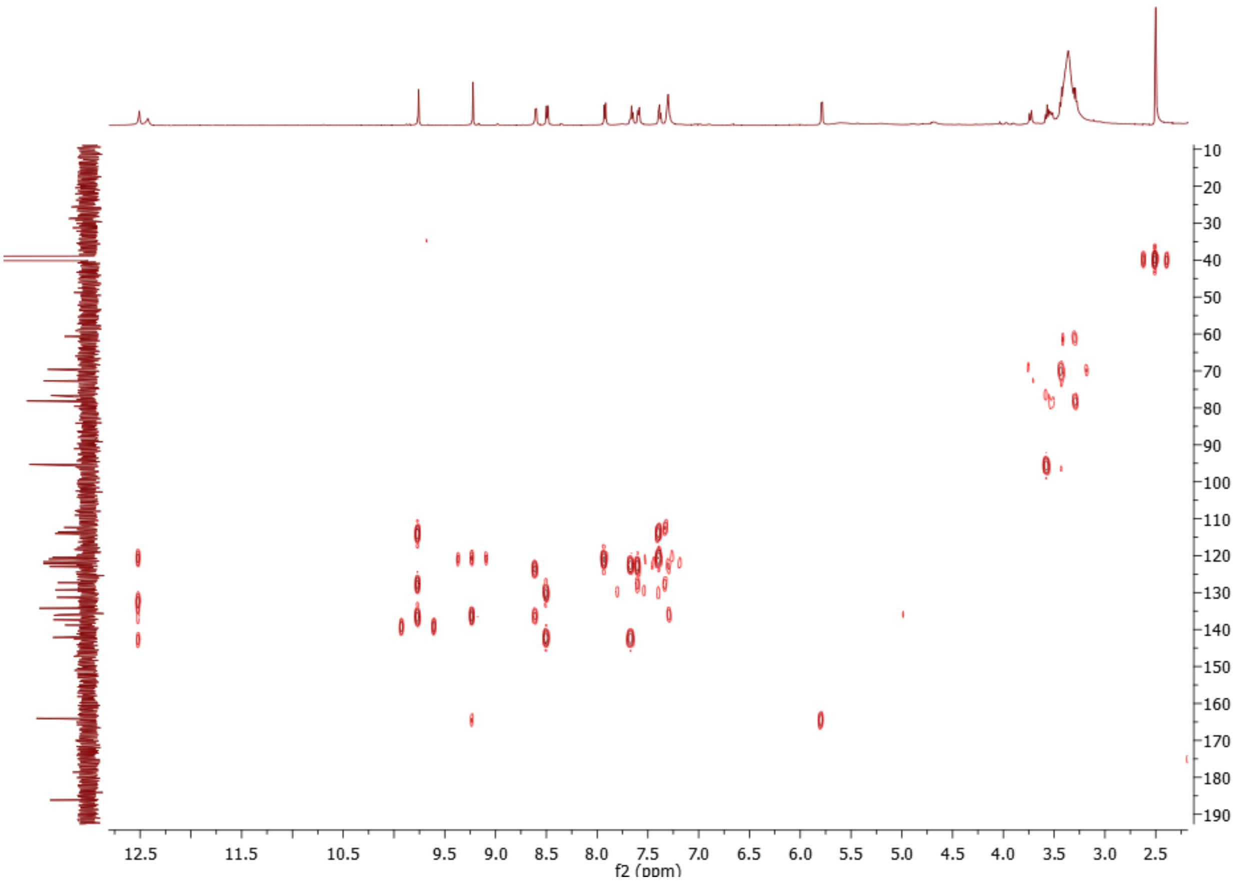

Figure S10. HMBC spectrum of **1** (DMSO-*d6*).

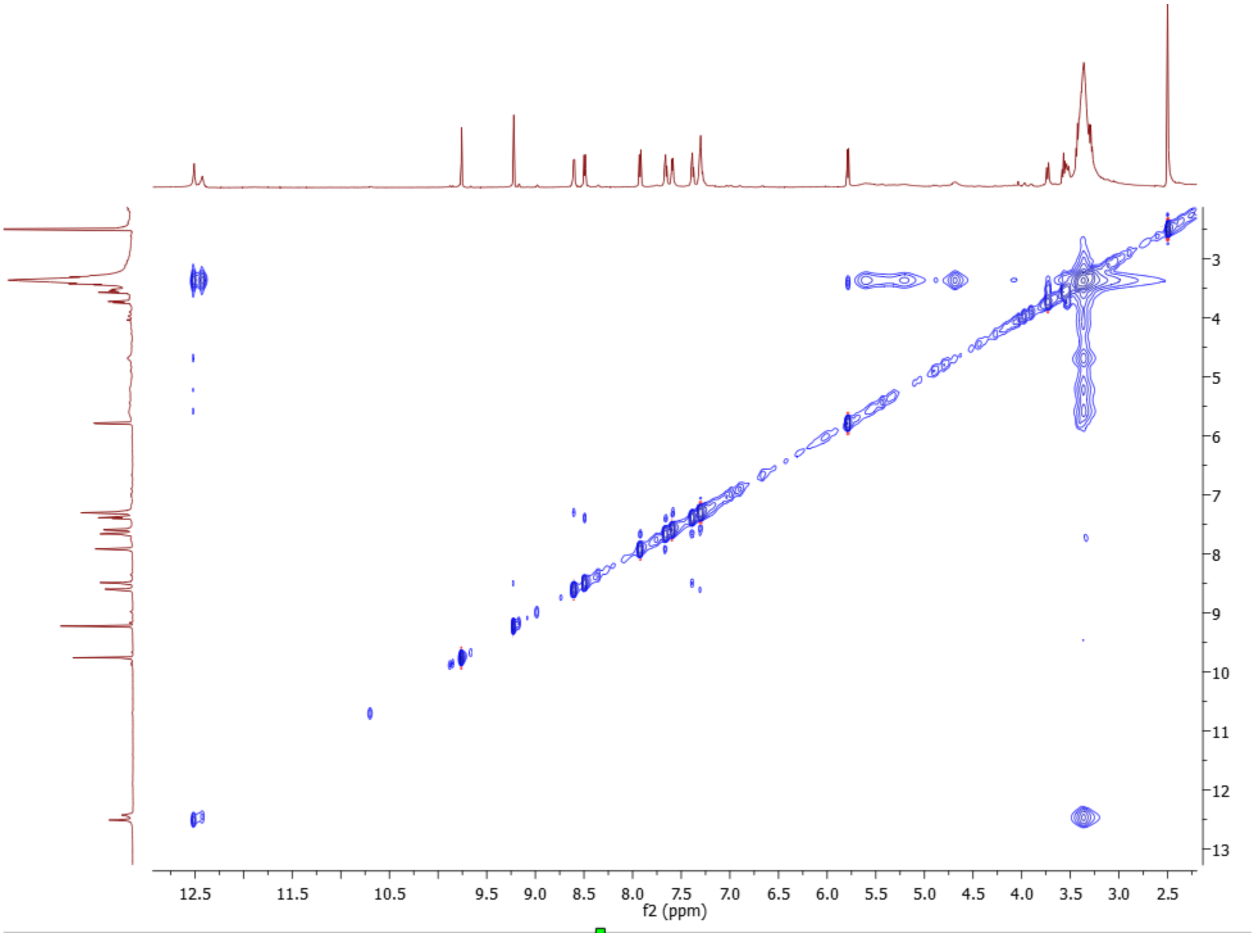

Figure S11. NOESY spectrum of **1** (DMSO-*d6*).

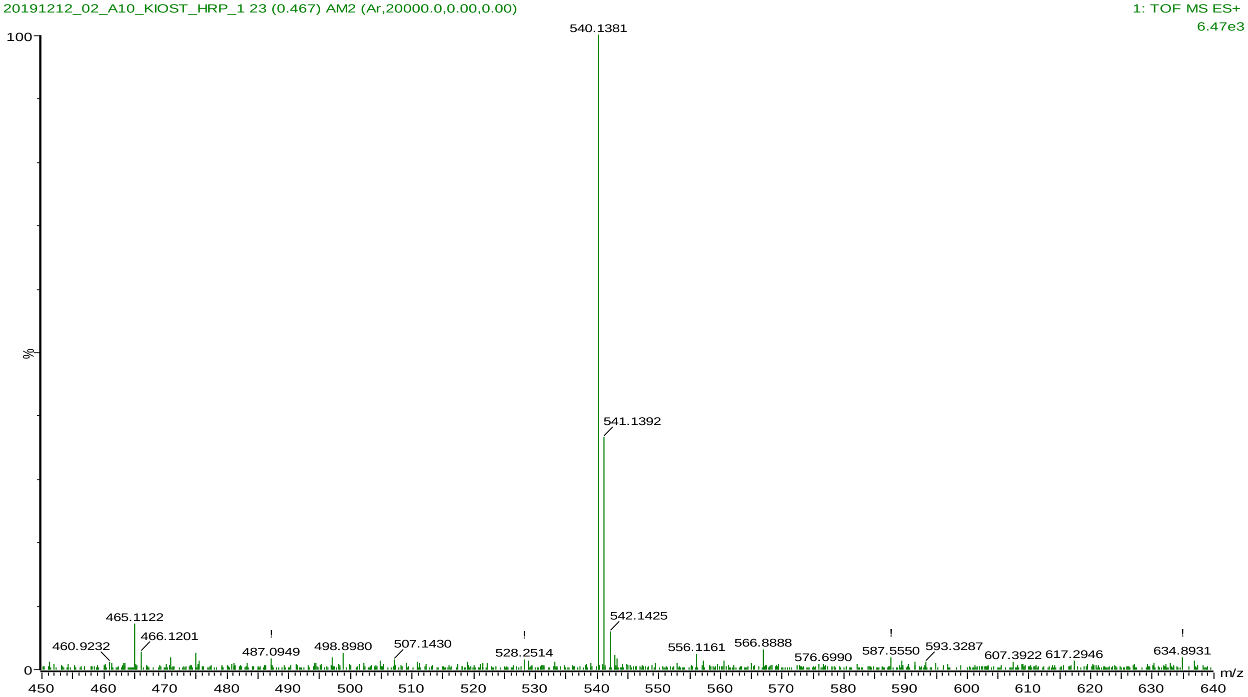

Mass	Calc. Mass	mDa	PPM	DBE	i-FIT	Norm	Conf(%)	Formula
540.1381	540.1383	-0.2	-0.4	17.5	75.4	n/a	n/a	C27 H23 N3 O8 Na

Figure S12. HRESIMS data of **1**.

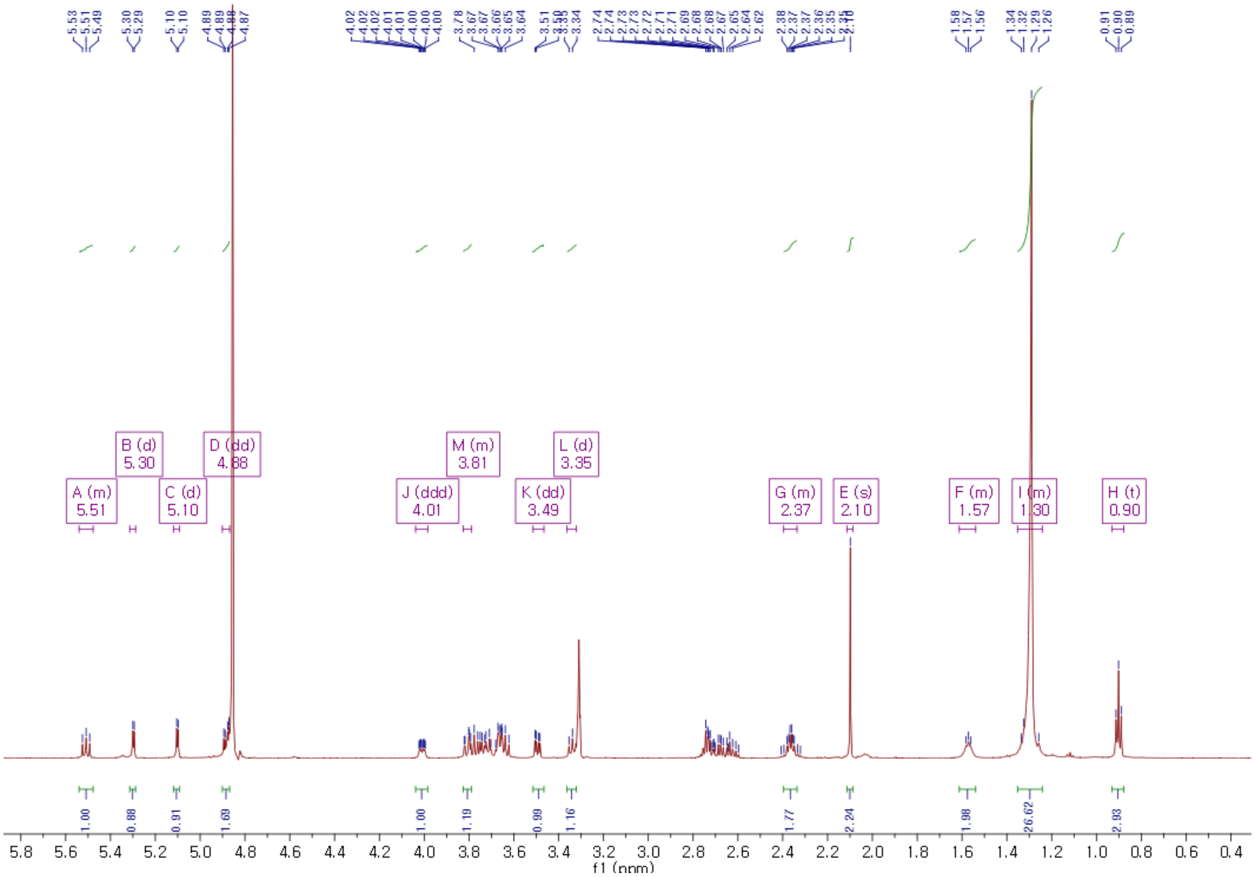

Figure S13. ^{1}H NMR spectrum of **2** (CD_3OD, 600 MHz).

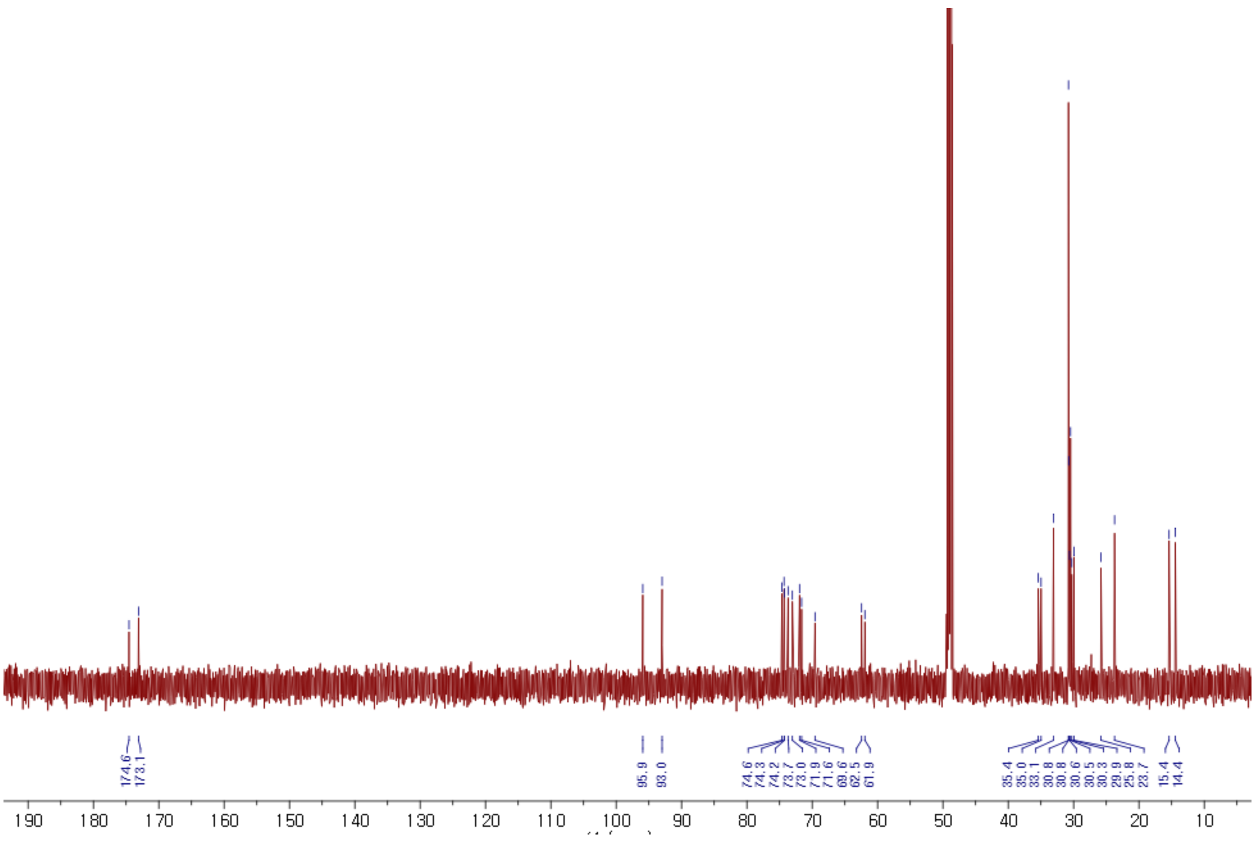

Figure S14. ^{13}C NMR spectrum of **2** (CD_3OD, 150 MHz).

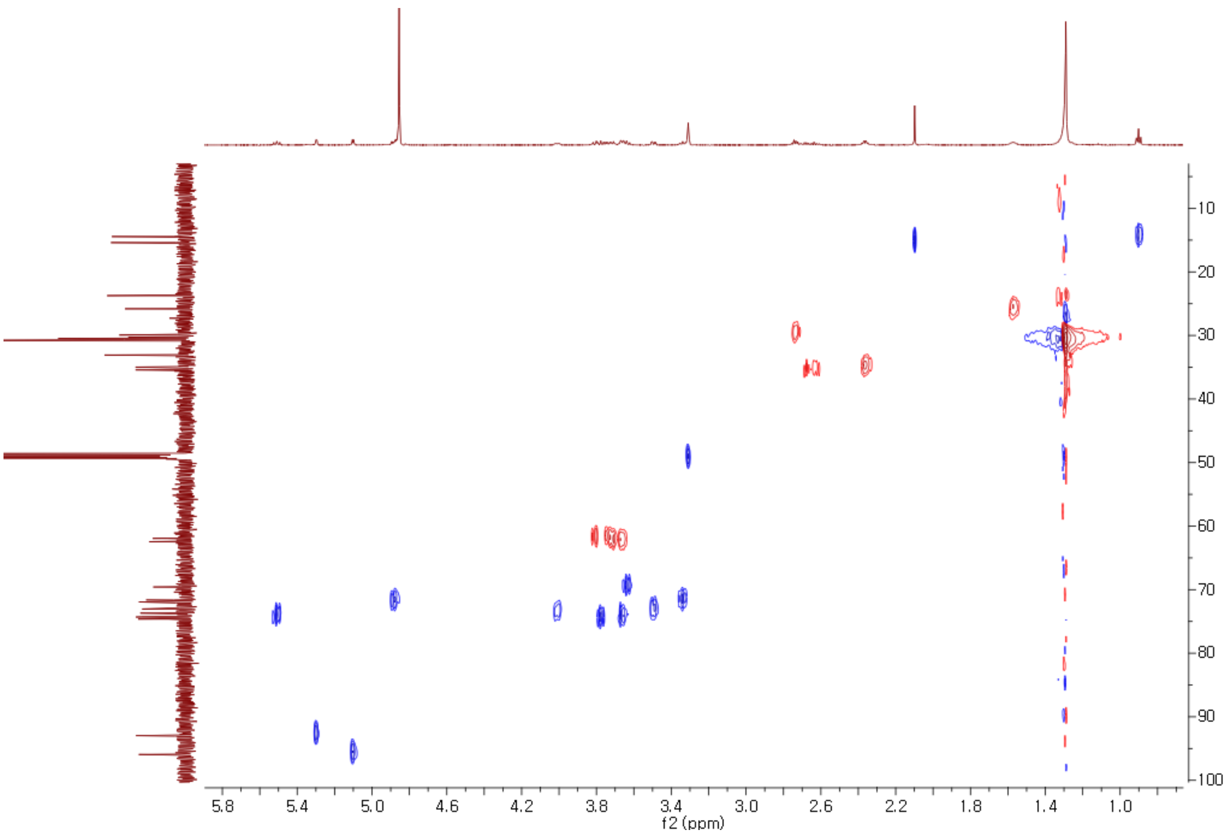

Figure S15. HSQC spectrum of **2** (CD_3OD).

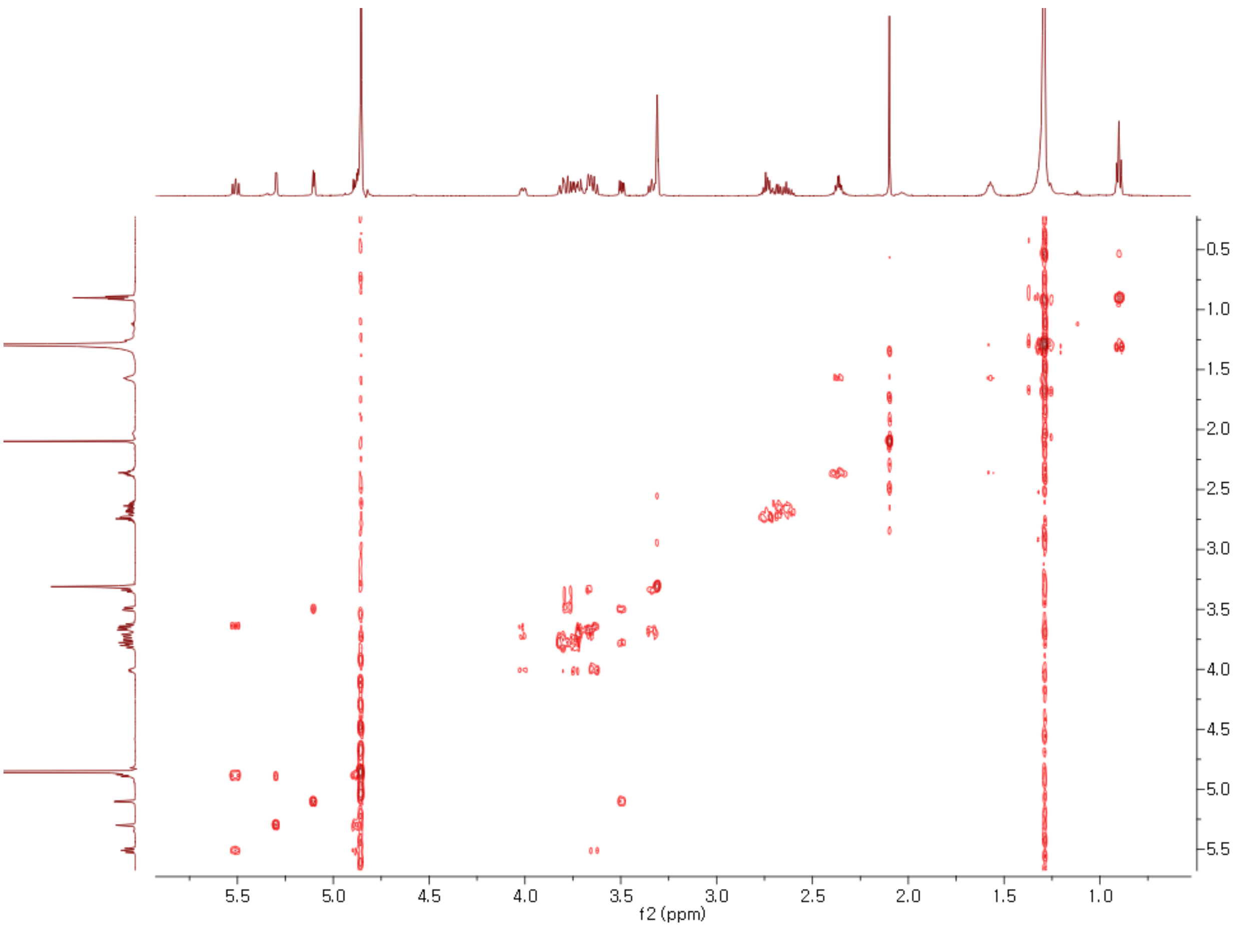

Figure S16. ^{1}H-^{1}H COSY spectrum of **2** (CD_3OD).

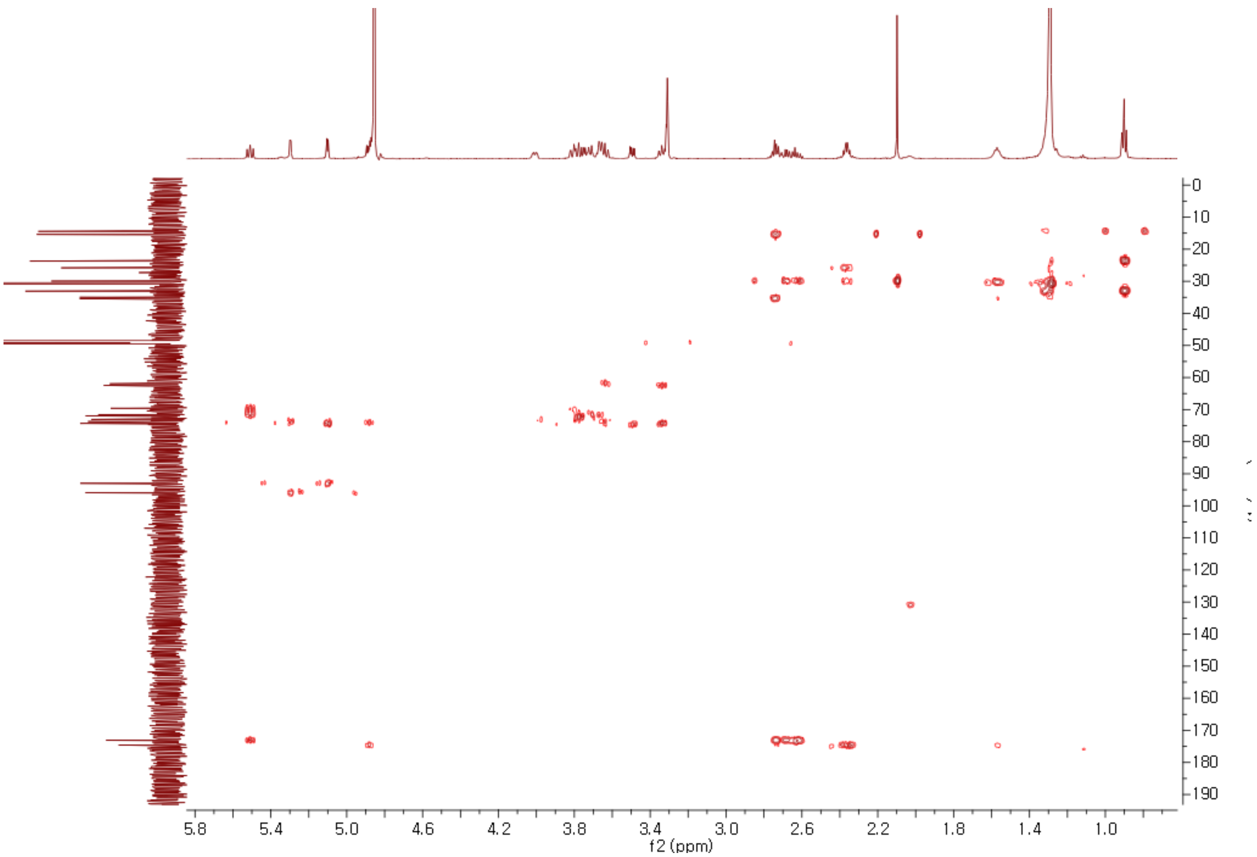

Figure S17. HMBC spectrum of **2** (CD_3OD).

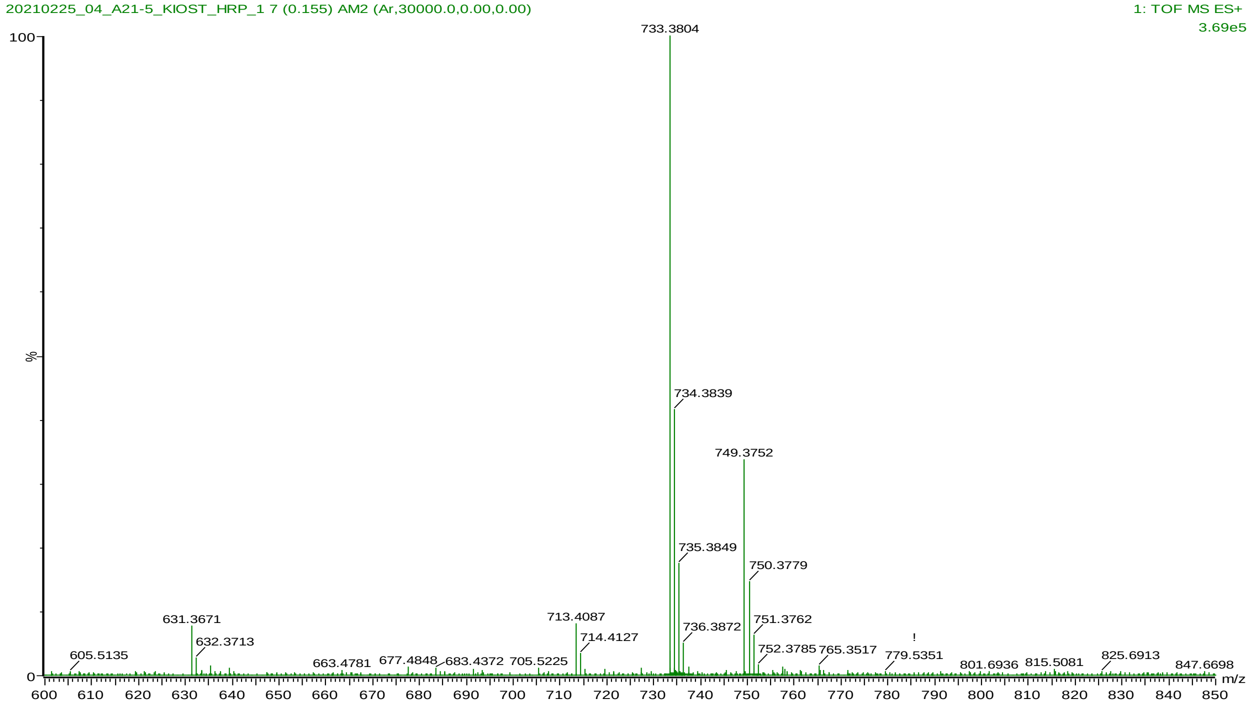

Mass	Calc. Mass	mDa	PPM	DBE	i-FIT	Norm	Conf(%)	Formula
733.3804	733.3809	-0.5	-0.7	3.5	580.1	n/a	n/a	C34 H62 O13 Na S

Figure S18. HRESIMS data of **2**.

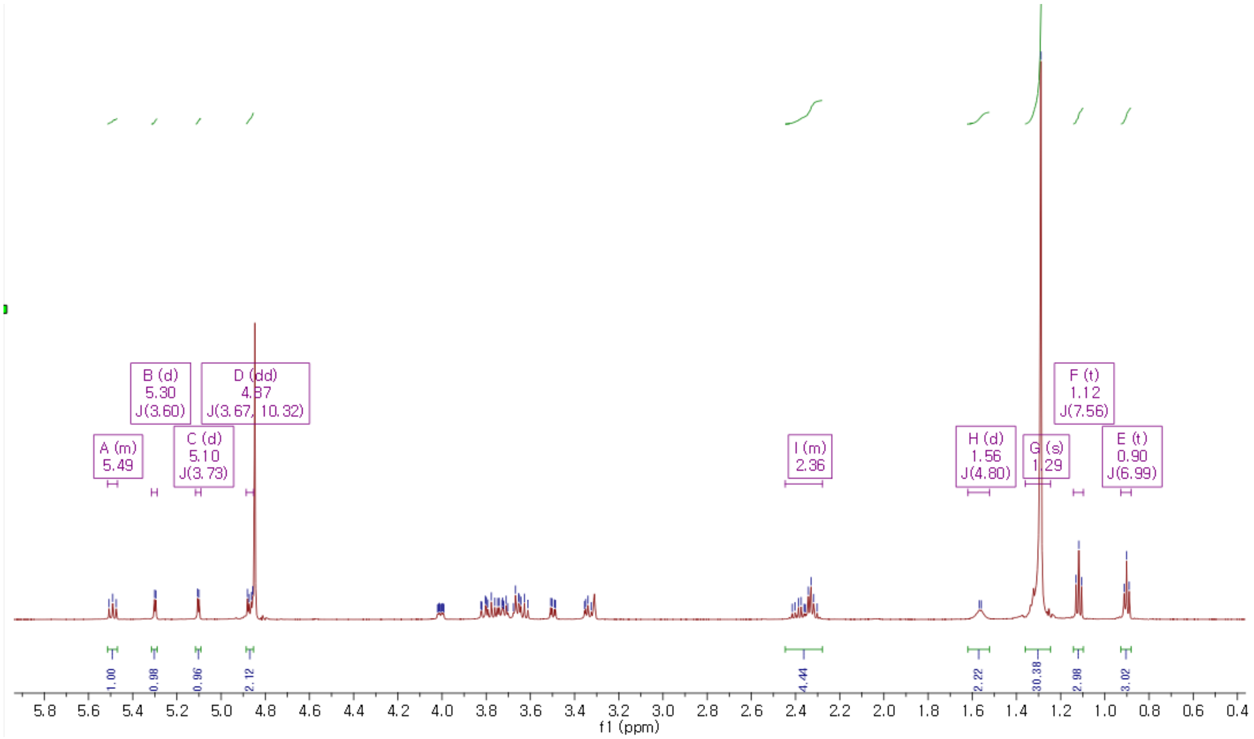

Figure S19. ^{1}H NMR spectrum of **3** (CD_3OD, 600 MHz).

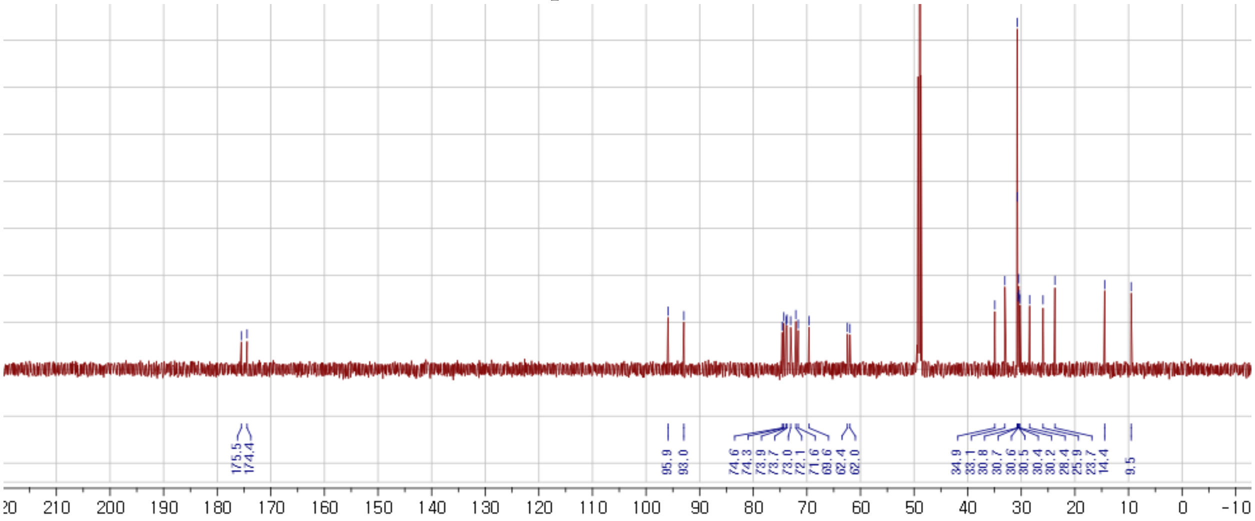

Figure S20. ^{13}C NMR spectrum of **3** (CD_3OD, 150 MHz).

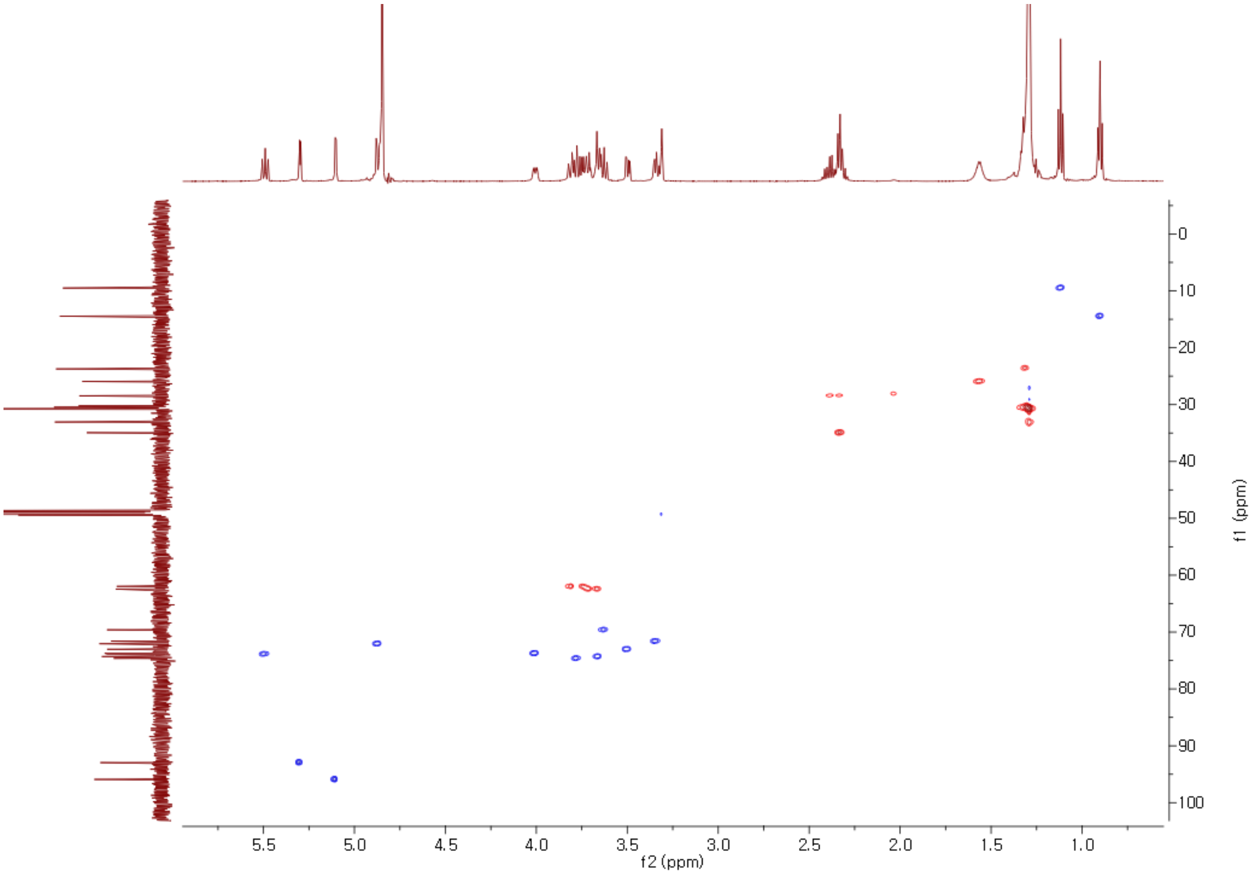

Figure S21. HSQC spectrum of **3** (CD_3OD).

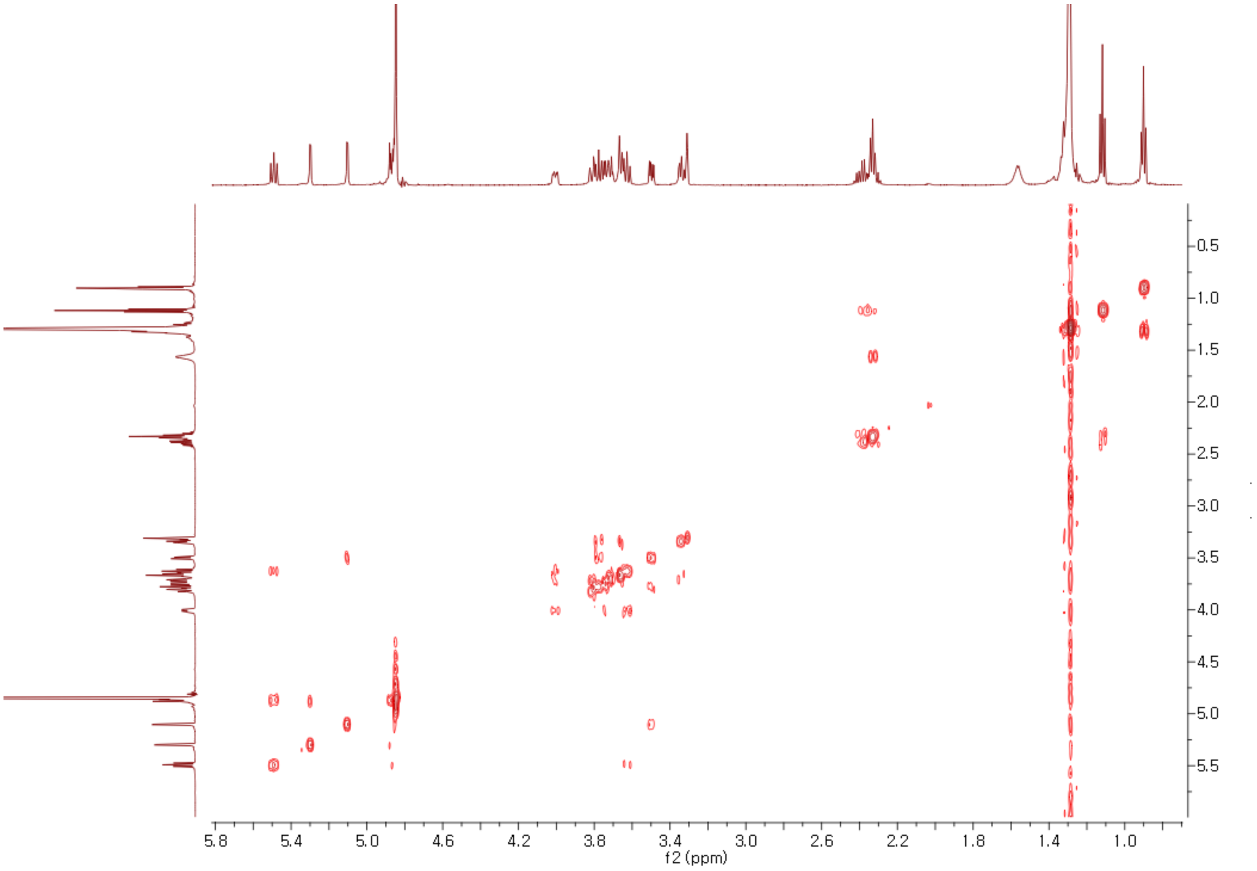

Figure S22. ^1H-^1H COSY spectrum of **3** (CD_3OD).

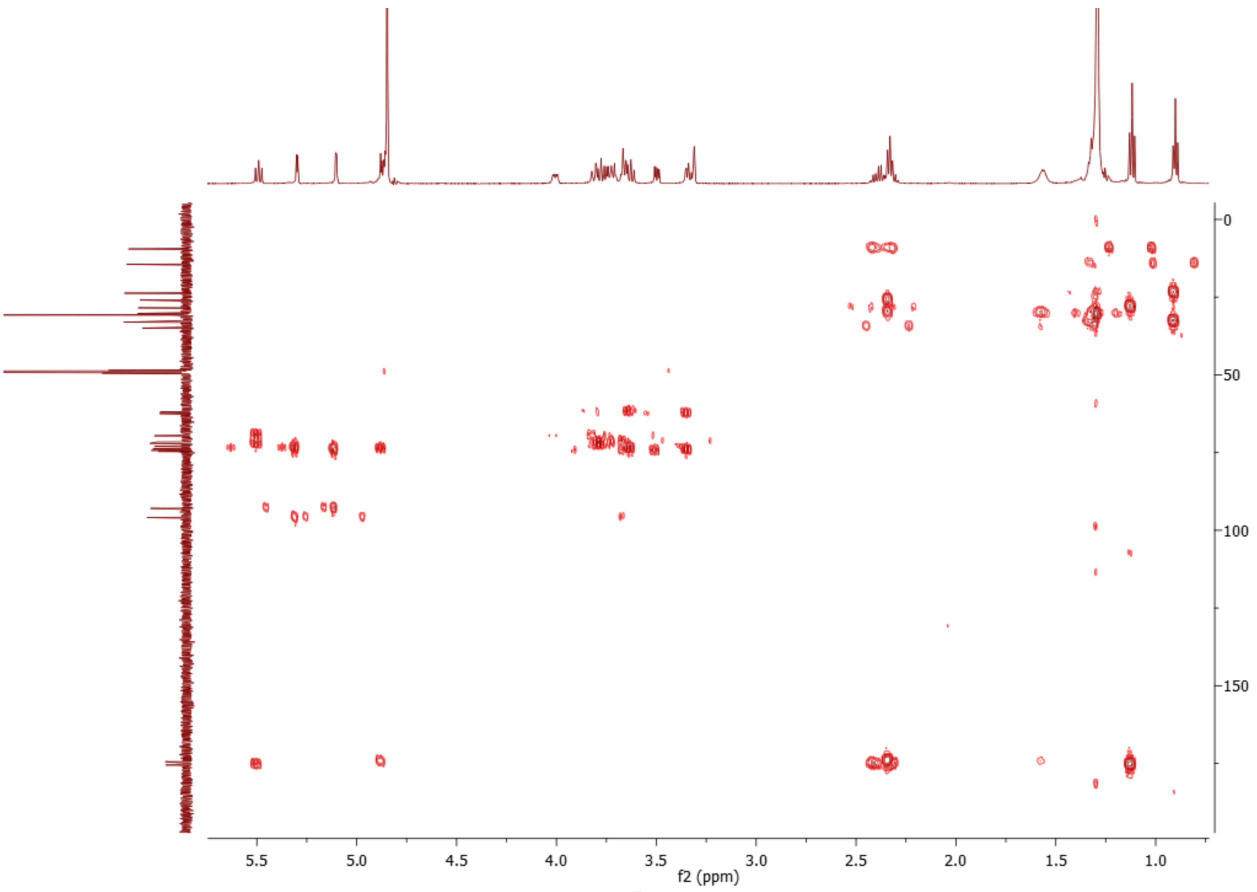

Figure S23. HMBC spectrum of **3** (CD_3OD).

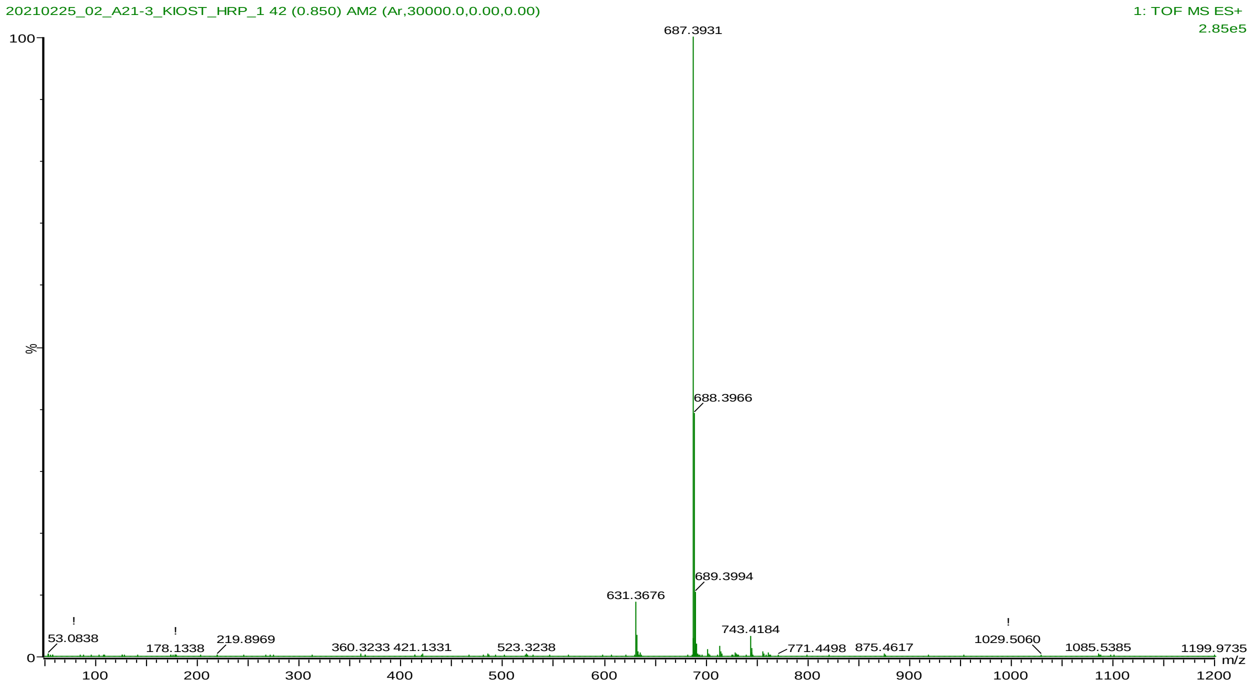

Mass	Calc. Mass	mDa	PPM	DBE	i-FIT	Norm	Conf(%)	Formula
687.3931	687.3932	-0.1	-0.1	3.5	604.8	n/a	n/a	C33 H60 O13 Na

Figure S24. HRESIMS data of **3**.

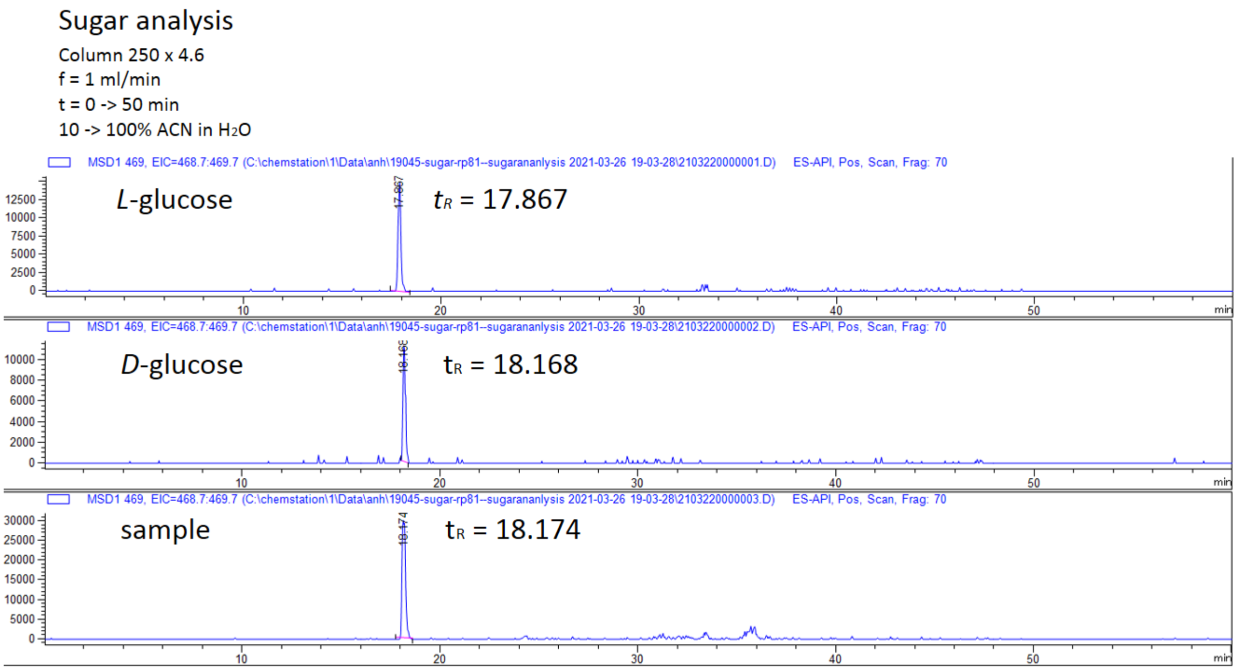

Figure S25. Analysis of sugar moieties.

NUGC-3

	1			Adriamycin	
Conc.(ug/ml)	Mean	SD	Conc.(ug/ml)	Mean	SD
30	-16.77	8.02	3	-14.45	7.71
10	28.63	6.86	1	-7.62	7.46
3	73.93	9.04	0.3	22.86	5.66
1	82.84	9.09	0.1	38.30	6.50
0.3	94.75	5.06	0.03	91.21	3.56
GI50	5.062		GI50	0.088	
GI50 μM	9.79			0.16	

NCI-H23

	1			Adriamycin	
Conc.(ug/ml)	Mean	SD	Conc.(ug/ml)	Mean	SD
30	-9.90	4.35	3	-16.77	7.55
10	26.41	5.72	1	-8.45	3.60
3	76.13	6.61	0.3	18.45	7.82
1	86.44	8.76	0.1	27.85	4.89
0.3	93.90	4.21	0.03	94.57	6.63
GI50	5.293		GI50	0.073	
GI50 μM	10.24			0.14	

HCT-15

	1			Adriamycin	
Conc.(ug/ml)	Mean	SD	Conc.(ug/ml)	Mean	SD
30	-11.58	10.53	3	-13.39	3.44
10	21.13	2.87	1	-0.42	5.46
3	82.62	3.27	0.3	11.15	3.39
1	91.65	5.78	0.1	25.53	7.97
0.3	100.32	2.57	0.03	93.14	3.06
GI50	5.618		GI50	0.070	
GI50 μM	10.87			0.13	

PC-3

	1			Adriamycin	
Conc.(ug/ml)	Mean	SD	Conc.(ug/ml)	Mean	SD
30	-11.94	5.95	3	-12.75	4.01
10	25.05	8.61	1	-3.61	2.60
3	70.41	7.68	0.3	24.99	4.83
1	81.93	8.80	0.1	38.72	5.68
0.3	94.75	3.25	0.03	96.51	6.20
GI50	4.547		GI50	0.094	
GI50 μM	8.79			0.17	

MDA-MB-231

	1			Adriamycin	
Conc.(ug/ml)	Mean	SD	Conc.(ug/ml)	Mean	SD
30	-13.23	6.64	3	-11.82	3.32
10	21.78	8.41	1	-2.04	5.54
3	67.98	7.89	0.3	17.73	6.55
1	81.05	6.60	0.1	38.89	3.59
0.3	93.04	2.64	0.03	90.63	5.03
GI50	4.157		GI50	0.086	
GI50 μM	8.04			0.16	

ACHN

	1			Adriamycin	
Conc.(ug/ml)	Mean	SD	Conc.(ug/ml)	Mean	SD
30	-12.29	7.98	3	-11.38	5.10
10	39.66	4.73	1	-3.93	3.20
3	67.85	6.68	0.3	25.42	4.90
1	83.53	2.69	0.1	36.65	8.65
0.3	97.09	2.25	0.03	94.22	2.84
GI50	5.219		GI50	0.090	
GI50 μM	10.09			0.17	

Figure S26. Results of the cytotoxicity test of **1**.

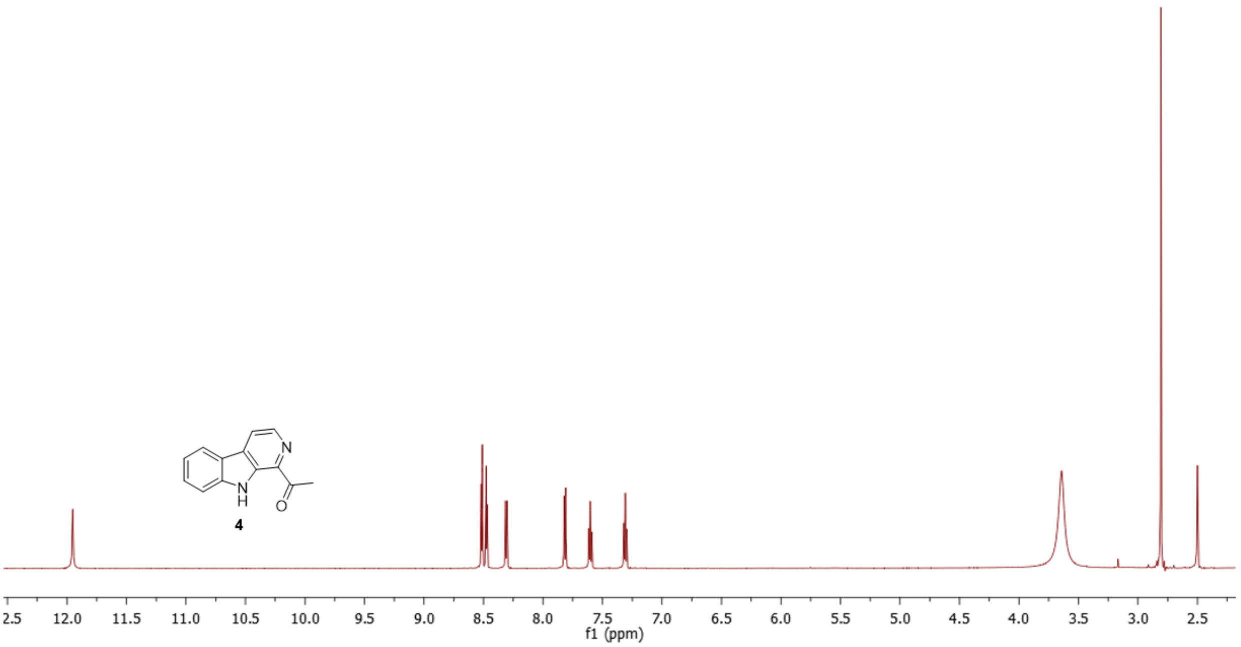

Figure S27. ^{1}H NMR spectrum of **4** (DMSO-d_6, 600 MHz).

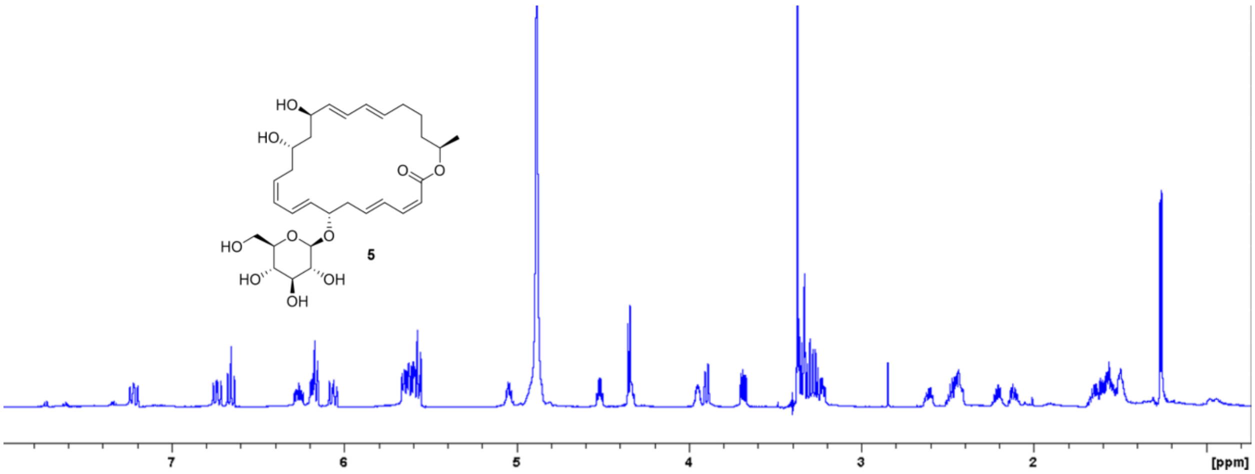

Figure S28. ^{1}H NMR spectrum of **5** (CD_3OD, 600 MHz).

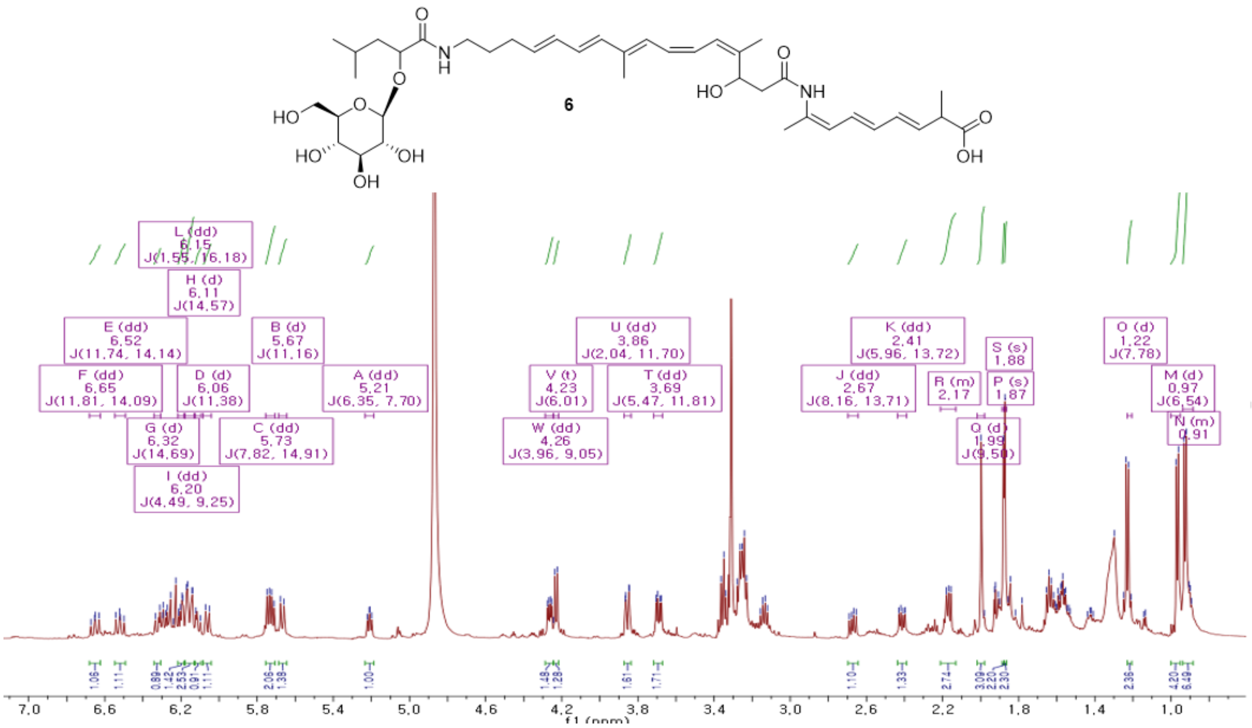

Figure S29. ^{1}H NMR spectrum of **6** (CD_3OD, 600 MHz).
